# Stabilization of pre-existing neurotensin receptor conformational states by β-arrestin-1 and the biased allosteric modulator ML314

**DOI:** 10.1038/s41467-023-38894-8

**Published:** 2023-06-07

**Authors:** Fabian Bumbak, James B. Bower, Skylar C. Zemmer, Asuka Inoue, Miquel Pons, Juan Carlos Paniagua, Fei Yan, James Ford, Hongwei Wu, Scott A. Robson, Ross A. D. Bathgate, Daniel J. Scott, Paul R. Gooley, Joshua J. Ziarek

**Affiliations:** 1 Department of Molecular and Cellular Biochemistry, Indiana University, Bloomington, IN 47405 USA; 2 Graduate School of Pharmaceutical Sciences, Tohoku University, Sendai, Miyagi 980-8578 Japan; 3 Biomolecular NMR laboratory, Department of Inorganic and Organic Chemistry, Universitat de Barcelona (UB), 08028 Barcelona, Spain; 4 Department of Materials Science and Physical Chemistry & Institute of Theoretical and Computational Chemistry (IQTCUB), Universitat de Barcelona (UB), 08028 Barcelona, Spain; 5 Department of Biochemistry and Pharmacology, Bio21 Molecular Science and Biotechnology Institute, University of Melbourne, Parkville, VIC 3010 Australia; 6 Department of Chemistry, Indiana University, Bloomington, IN 47405-7102 USA; 7 The Florey Institute of Neuroscience and Mental Health and Department of Biochemistry and Pharmacology, The University of Melbourne, Parkville, VIC 3010 Australia; 8 Present Address: ARC Centre for Cryo-electron Microscopy of Membrane Proteins and Drug Discovery Biology, Monash Institute of Pharmaceutical Sciences, Monash University, Parkville, VIC 3052 Australia; 9 Present Address: School of Chemistry & Biochemistry, Georgia Institute of Technology, Atlanta, GA 30332 USA

**Keywords:** Molecular conformation, G protein-coupled receptors, Solution-state NMR

## Abstract

The neurotensin receptor 1 (NTS_1_) is a G protein-coupled receptor (GPCR) with promise as a drug target for the treatment of pain, schizophrenia, obesity, addiction, and various cancers. A detailed picture of the NTS_1_ structural landscape has been established by X-ray crystallography and cryo-EM and yet, the molecular determinants for why a receptor couples to G protein versus arrestin transducers remain poorly defined. We used ^13^C^ε^H_3_-methionine NMR spectroscopy to show that binding of phosphatidylinositol-4,5-bisphosphate (PIP2) to the receptor’s intracellular surface allosterically tunes the timescale of motions at the orthosteric pocket and conserved activation motifs – without dramatically altering the structural ensemble. β-arrestin-1 further remodels the receptor ensemble by reducing conformational exchange kinetics for a subset of resonances, whereas G protein coupling has little to no effect on exchange rates. A β-arrestin biased allosteric modulator transforms the NTS_1_:G protein complex into a concatenation of substates, without triggering transducer dissociation, suggesting that it may function by stabilizing signaling incompetent G protein conformations such as the non-canonical state. Together, our work demonstrates the importance of kinetic information to a complete picture of the GPCR activation landscape.

## Abstract (web summary)

The authors highlight the importance of kinetics in GPCR activation. Using solution NMR, they show that the transducer βarrestin1 and the β-arrestin1biased ligand ML314 kinetically tune the conformational ensemble of the neurotensin receptor 1.

## Introduction

Cells rely on membrane-embedded receptors to maintain awareness of the extracellular environment without compromising membrane integrity. The G protein-coupled receptor (GPCR) superfamily is the largest group among such eukaryotic cell surface receptors, comprising more than 800 proteins^1,2^. They are ubiquitously expressed throughout the human body and are pivotal in a broad range of physiological processes including vision, taste, sense of smell, nervous functions, immune regulation, reproduction, and cancer^3,4^. Ligand binding at the extracellular, orthosteric site allosterically induces conformational changes across the GPCRs’ signature seven transmembrane (7TM) helices that prime the intracellular face for interaction with transducer proteins such as G proteins, β-arrestins (βArr), and G protein-coupled receptor kinases (GRKs)^5^. The neurotensin receptor 1 (NTS_1_) is a high-affinity target for the endogenous 13-residue peptide agonist neurotensin (NT)^6^. NT functions as a neuromodulator of the central nervous system (CNS) as well as a paracrine and endocrine modulator of the digestive tract and cardiovascular system^7^.

The strong expression overlaps of the dopamine system with both NT and NTS_1_ has led to considerable evidence for functional synergy in psychostimulant and opioid drug addiction^8–12^. Despite the long-standing interest in NTS_1_ as a potential therapeutic target for substance use disorders (SUDs), the handful of small-molecule NTS_1_ agonists and antagonists that have been developed all suffer from on-target side effects such as hypothermia^13,14^, hypotension^15^, and impaired motor control^15,16^. The classical model of GPCR activation implies that ligand-bound receptors signal equally (aka balanced) through G protein and β-arrestin (βArr) transducer pathways. The recent recognition of biased signaling, in which ligands preferentially activate one transducer pathway over the other, offers a new treatment avenue that may reduce on-target side effects^17–19^. A high-throughput functional screen and ligand optimization campaign targeting NTS_1_ led to the development of ML314, an allosteric ligand that selectively activates βArr2 pathways without stimulating the Gq pathway (i.e. βArr2-biased allosteric modulator; BAM), which reduces addictive behaviors toward methamphetamine and cocaine in several mouse models^20,21^. ML314 also functions as a βArr1-BAM^22^.

Yet, the molecular determinants for why a ligand promotes G protein:receptor versus arrestin:receptor complexation remain poorly defined. The predominant hypothesis is that agonists stabilize distinct receptor conformations to preferentially activate one pathway over another. Recent cryo-EM structures of NTS_1_:βArr1 and NTS_1_:G protein, however, reveal a remarkably conserved receptor architecture with a 0.67 Å all-atom RMSD^23–26^. Solution NMR has proven indispensable for identifying receptor conformers that are beyond the resolution of static structural methods^27^, but few studies have investigated G protein^28–30^ or βArr^31–33^ ternary complexes; to date, the only GPCRs characterized by NMR in complex with mimetics of both transducers are NTS_1_^33^, M2 muscarinic^34^, and the β2-adrenergic receptor^31,32,35,36^. Here, we uniformly-label ^13^C^ε^H_3_-methionine residues located within the NTS_1_ transmembrane bundle and near the ligand-binding site to demonstrate how ligands and PIP2 dynamically prepare the receptor for transducer interaction. The differential conformational kinetics upon coupling to βArr and G protein transducer molecules suggest a role for dynamics in functional selectivity.

## Results

### PIP2 strengthens correlated motions of the orthosteric pocket and PIF motif

**Fig. 1:**
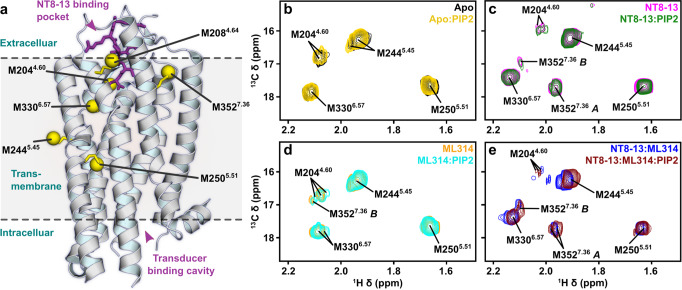
Effect of PIP2 on enNTS_1_ΔM4 ^13^C^ε^H_3_-methionine chemical shifts. **a** Cartoon representation of thermostabilized rNTS_1_ (PDB 4BWB) with labelled methionine methyl groups shown as yellow spheres (superscript - Ballesteros-Weinstein nomenclature^41^) and NT8-13 shown as purple sticks. Overlays of Apo-state (**b**), NT8-13 (**c**), ML314 (**d**), and NT8-13 & ML314 (**e**) bound ^1^H-^13^C HMQC spectra in the absence and presence of 130 μM (2x molecular equivalents over enNTS_1_) PIP2. The corresponding peak intensities are plotted in Supplemental Fig. S1. All spectra were recorded at 600 MHz, in 3 mm thin wall precision NMR tubes (Wilmad), with enNTS_1_ΔM4 concentrations of 66 μM.

Phosphatidylinositol-4,5-bisphosphate (PIP2), and its analog (C8-PIP2; here termed PIP2), enhance both G protein activation^37,38^ and arrestin recruitment^23^. A NTS_1_:βArr1 cryo-EM complex includes one PIP2 molecule bound in a pocket formed by the intracellular portions of TMs 1/2/4^23^; comparison with both canonical and non-canonical NTS_1_:G protein cryo-EM models suggests this PIP2 position could also coordinate the interaction of NTS_1_ with the Gαβ subunits^24,25^. Here, we used a previously characterized minimal methionine enNTS_1_ variant (herein enNTS_1_ΔM4)^22^ to test if PIP2 directly affects the receptor’s conformational ensemble in the absence of transducers. enNTS_1_ΔM4 was derived from a thermostabilized rat NTS_1_ (rNTS_1_) variant (enNTS_1_)^39,40^ by removing four of the ten endogenous methionines to eliminate signal overlap in NMR spectra. The six remaining residues (M204^4.60^, M208^4.64^, M244^5.45^, M250^5.51^, M330^6.57^, and M352^7.36^; superscript refers to Ballesteros-Weinstein numbering^41^) are located at the extracellular region, the base of the orthosteric pocket, and the PIF motif (Fig. 1a). Mutation of the four methionine residues did not adversely affect enNTS_1_’s structural integrity or function^22^. Two-dimensional (2D) ^1^H-^13^C heteronuclear multiple quantum correlation (HMQC) spectra were collected for apo, NT8-13 bound, ML314 bound, and NT8-13:ML314 bound [^13^C^ε^H_3_-methionine]-enNTS_1_ΔM4 in the presence and absence of PIP2 (Fig. 1b–e). NT8-13 is an orthosteric ligand comprised of NT’s six C-terminal amino acids and is sufficient to produce full agonist activity^42^. ML314 is classified as a biased allosteric modulator (BAM) because it binds an alternative unknown site to potentiate NT8-13-mediated βArr1 recruitment while simultaneously attenuating agonist-induced G protein activation^22^. ML314 was reported to selectively stimulate βArr2 recruitment independent of NT8-13^20,21^, a pharmacological classification known as an agonist (ago)-BAM, although this was not observed in enNTS_1_ΔM4 or wildtype human NTS_1_ functional assays^22^.

**Fig. 2:**
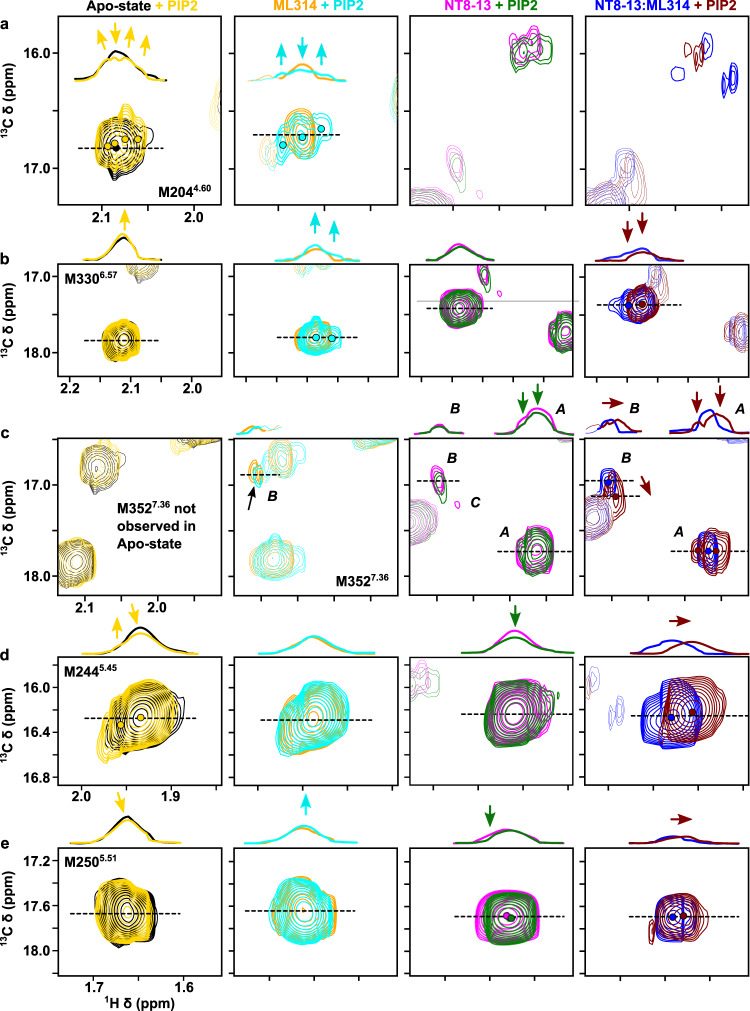
Effect of PIP2 on enNTS_1_ΔM4 ^13^C^ε^H_3_-methionine chemical shifts. Expanded views of individual resonances shown in Fig. 1. M204^4.60^ (**a**), M330^6.57^ (**b**), M352^7.36^ (**c**), M244^5.54^ (**d**), and M250^5.51^ (**e**) in the absence/presence of 130 μM (2× molecular equivalents over enNTS_1_) PIP2 for Apo-state (black/yellow), ML314-bound (orange/cyan), NT8-13-bound (magenta/dark green), and NT8-13:ML314-bound (blue/brown) enNTS_1_. The resonances of other residues within the extracted region are drawn at 50% transparency. The ^1^H 1D traces given above each spectrum correspond to the dashed lines indicated in each 2D spectrum below. All spectra were recorded at 600 MHz, in 3 mm thin wall precision NMR tubes (Wilmad), with enNTS_1_ΔM4 concentrations of 66 μM.

All NMR spectra were collected at 66 μM [^13^C^ε^H_3_-methionine]-enNTS_1_ΔM4 with identical acquisition, processing, and display parameters; thus, we can directly compare both the chemical shift values (i.e. structure) and signal intensities (i.e. dynamics) for each liganded state. Surprisingly, except for the NT8-13:ML314 bound state, PIP2 induced only subtle chemical shift perturbations indicating minimal effect on conformer populations (Figs. 1 and 2). However, when both agonist and BAM are present, PIP2 perturbs the M204^4.60^, M244^5.45^, and M250^5.51^ chemical shifts (i.e. pushes the structural equilibrium) towards the NT8-13 bound state (Figs. 1e and 2a, d, e). These chemical shift changes could reflect population changes towards more balanced NTS_1_:transducer complexes^23,37,38^, negative cooperativity between PIP2 and ML314 sites, or even direct binding competition.

For the other liganded states, PIP2 primarily alters peak shapes and/or intensities, which reflect changes in receptor dynamics on the slow (ms-s) and fast-intermediate (ps-ms) timescales, respectively. In several instances, such as apo or ML314 bound M204^4.60^, a single uniform resonance develops multiplet character without adopting a new chemical shift value (Fig. 2a). The simplest explanation for this behavior is that the methyl group was rapidly exchanging between two or more distinct environments, and PIP2 slowed the interconversion rate without modifying the underlying nature of each conformer. Multiple NT8-13:ML314 bound resonances exhibit the opposite phenomena with peaks coalescing as PIP2 accelerates the rate of conformational exchange (Fig. 2b, d, e). In other cases, such as ML314-bound M330^6.57^ or apo M244^5.45^, PIP2 amends the relative intensity (i.e. equilibrium populations) of multiplet components (Figs. 2b, d and S1). Interestingly, PIP2 remodels the apo M244^5.45^ doublet intensity to match that of the M314-bound state, yet addition of PIP2 to ML314:enNTS_1_ΔM4 has no additional effect (Fig. 2d). Given that PIP2 may bind NTS_1_ at multiple locations, this may suggest partially overlapping interfaces or negative cooperatively between sites. A loss in peak intensity is typically caused by line broadening that may signify either changes in the intrinsic transverse relaxation rate (R_2_) and/or exchange broadening (R_ex_). R_2_ relaxation results from physical properties of the methyl group on the ps-ns timescale, while R_ex_ reflects conformational interconversion in the μs-ms regime^43^. In the apo state, PIP2 sharpens M330^6.57^ adjacent to the ligand-binding pocket even as it broadens M204^4.60^ at the base of the pocket and M250^5.51^ of the connector region (Figs. 1b, 2, and Supplementary Fig. 1). The NT8-13 bound receptor resonances are universally broadened by PIP2 (Figs. 1c and 2 and Supplementary Fig. 1) whereas the ML314:enNTS_1_ΔM4 complex appears rigidified in the extracellular region (M330^6.57^), near the base of the orthosteric pocket (M204^4.60^), and surrounding the PIF motif (M250^5.51^; Figs. 1d and 2 and Supplementary Fig. 1).

The M352^7.36^ peak pattern in both NT8-13:enNTS_1_ΔM4 (Figs. 1c and 2c) and NT8-13:ML314:enNTS_1_ΔM4 (Figs. 1e and 2c) complexes reflects a multi-state equilibrium with mixed exchange regimes^44^. At first approximation, the presence of three peaks (states A, B, and C) indicates that M352^7.36^ exchanges between three chemical environments qualitatively on the slow (i.e. ms-s) timescale^22,45^. Density functional theory (DFT)-guided NMR analysis^22^ suggests that state A represents tight packing of TM1/6/7 and lid-like engagement of the N-terminus against the bound NT8-13 as observed in the NTSR1-H4_X_:NT8-13 X-ray structure (PDB 6YVR^46^), whereas M352^7.36^*B* may reflect detachment of the receptor N-terminus and local stabilization of extracellular TM1/6/7 as observed in the NTSR1-H4_X_:SR142948A structure (PDB 6Z4Q^46^). In the agonist bound state, PIP2 modestly reduced the total (sum of states A and B) M352^7.36^ peak intensity by 22.6%, which suggests a subtle adjustment towards a faster state A to state B interconversion rate although still within the ms-s timescale^45^. At the same time, the relative M352^7.36^*B* population increased by 138.8% while the M352^7.36^*A* state was effectively unchanged (Figs. 1c and 2c, and Supplementary Fig. 1). One potential explanation for this behavior is that state B is a composite of two microstates exchanging on the intermediate-fast (μs-ms) timescale. When both agonist and BAM are present, PIP2 increases the total (sum of states A and B) M352^7.36^ peak intensity by 164% without changing the relative state A and B populations (66 and 34%, respectively), which would correspond to a reduction of the A to B interconversion rate on the slow (ms-s) timescale^45^. The M352^7.36^*B* resonance is simultaneously perturbed upfield in the ^1^H dimension and downfield in the ^13^C dimension (Figs. 1e and 2c). There are several possible explanations for this behavior: (i) a change in the relative populations of fast-exchanging (μs-ms) microstates that comprise state B, (ii) structural changes of the M352^7.36^*B* chemical environment itself, and (iii) remodeling of both the thermodynamic and kinetic properties of states A and B. Taken together, PIP2 clearly remodels the receptor’s structural and kinetic ensemble. To better understand the nature of PIP2-mediated spectra changes, we investigated complexes of enNTS_1_ΔM4 with arrestin and G protein mimetics. For the remainder of this study, unless otherwise stated, all samples include PIP2.

### βArr1 alters the kinetic landscape of the NTS_1_ conformational ensemble

**Fig. 3:**
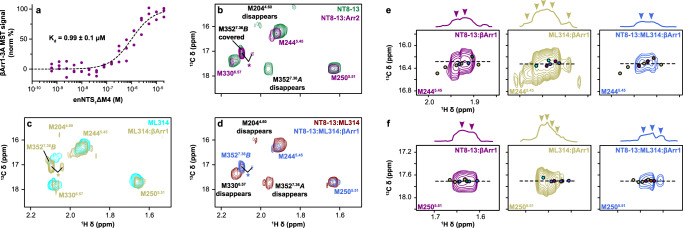
βArr1 stabilizes pre-existing states in the presence of agonist and/or BAM. **a** Microscale thermophoresis (MST) measured the affinity of βArr1-3A for NT8-13:enNTS_1_ΔM4 as 0.99 ± 0.1 μM ( ± SEM) using a single-site quadratic binding model. Data was collected as n = 3 biologically independent experiments with 5 or 6 technical repeats. Source data are provided as a Source Data file. Overlays of (**b**) NT8-13:enNTS_1_ΔM4 (forest green) and NT8-13:enNTS_1_ΔM4:βArr1-3A (purple), (**c**) ML314:enNTS_1_ΔM4 (cyan) and ML314:enNTS_1_ΔM4:βArr1-3A (tan), and (**d**) NT8-13:ML314:enNTS_1_ΔM4 (maroon) and NT8-13:ML314:enNTS_1_ΔM4:βArr1-3A (royal blue) ^1^H-^13^C HMQC spectra. Transducer spectra included 2.3x molar equivalents βArr1-3A. Peaks marked with an asterisk represent natural abundance βArr1-3A M411 (also see Supplementary Fig. 2b). Extracted spectral region of (**e**) M244^5.45^ and (**f**) M250^5.51^ from NT8-13:enNTS_1_ΔM4:βArr1-3A (purple), ML314:enNTS_1_ΔM4:βArr1-3A (tan), and NT8-13:ML314:enNTS_1_ΔM4:βArr1-3A (royal blue) ^1^H-^13^C HMQC spectra. One dimensional ^1^H cross-sectional slices (corresponding to dotted line) shown on top. Dots denote the residue’s chemical shift position in spectra of the corresponding colour with additional dots shown for ligand-only spectra (NT8-13:enNTS_1_ΔM4, forest green; ML314:enNTS_1_ΔM4, cyan; NT8-13:ML314:enNTS_1_ΔM4, maroon). All spectra were recorded at 600 MHz with receptor concentrations of 66 μM.

Recent cryo-EM structures of the NTS_1_:βArr1 complex required either protein fusion^26^ or intermolecular cross-linking^23^ to stabilize intrinsic dynamics, suggesting that NMR could provide additional information on the nature of these underlying motions. We utilized the pre-activated βArr1-3A variant to maximize the affinity for unphosphorylated enNTS_1_ΔM4^47,48^. Microscale thermophoresis (MST) measured the affinity of βArr1-3A for the NT8-13:enNTS_1_ΔM4 complex as 0.99 ± 0.1 μM, which corresponds to >98% receptor occupancy at a 2:1 βArr1-3A:enNTS_1_ΔM4 when assuming a single-site ligand depletion binding model (Fig. 3a). 2D transverse relaxation optimized spectroscopy (TROSY)-based ^1^H-^15^N heteronuclear single quantum correlation (HSQC) spectra of [*U*-^2^H,^13^C,^15^N]-βArr1-3A at increasing enNTS_1_ΔM4 concentrations confirm the formation of a specific complex (Supplementary Fig. 2a). The 2D ^1^H-^13^C HMQC of [^13^C^ε^H_3_-methionine]-enNTS_1_ΔM4:βArr1-3A ternary complex formation resulted in the appearance of two additional methionine peaks (Fig. 3b; asterisks). Collecting another ^1^H-^13^C HMQC spectrum using both unlabeled enNTS_1_ΔM4 and βArr1-3A revealed that both peaks belong to βArr1-3A; while the major resonance is always detectable, the minor peak is only visible in the presence of the receptor (Supplementary Fig. 2b). Neither resonance is observable when the experiment is repeated using βArr1 C-terminally truncated after N382 (βArr1-ΔCT^48^). As arrestin recruitment requires displacement of its self-associated C-tail^26,49^, we conclude that the major and minor resonances correspond to βArr1’s C-terminal M411 in the bound-basal and dissociated receptor-bound (or post-receptor-bound) state, respectively. In the absence of PIP2, βArr1-3A promotes limited [^13^C^ε^H_3_-methionine]-enNTS_1_ΔM4 spectral changes further supporting the lipid’s role in high affinity transducer complexation (Supplementary Fig. 2c).

βArr1-3A leads to exchange broadening of M204^4.60^, M352^7.36^ state A and presumably M352^7.36^ state B, although the latter is overlapped with the major βArr1-3A M411 resonance (Fig. 3b; asterisks). To better understand the chemical exchange kinetics, we performed a titration series with increasing βArr1-3A concentrations added to separate, otherwise identical, NT8-13:enNTS_1_ΔM4 samples (Supplementary Fig. 3). The intensity of every resonance decreased in a concentration-dependent manner reflecting the ternary complex’s longer rotational correlation time. The especially rapid broadening of M352^7.36^*A* suggests that arrestin back-coupling enhances μs-ms exchange kinetics at the periphery of the orthosteric pocket (Supplementary Fig. 3); this is consistent with the lack of density for the homologous hNTS_1_ M346^7.36^ sidechain in both NT8-13:hNTS_1_:βArr1 cryo-EM models^23,26^. The three methionines nearest to the transducer interface (M204^4.60^, M244^5.45^, and M250^5.51^) all split into at least two distinct conformational states exchanging on the ms-s timescale (Supplementary Fig. 3g–i). The major M244^5.45^ peak settled at a chemical shift position linearly between the apo- and NT8-13 bound states (Supplementary Fig. 3h) with a second peak produced at a similar chemical shift observed for the apo- and ML314 bound states (Fig. 1b, d). βArr1-3A splits M250^5.51^ into two peaks centered at the NT8-13-bound chemical shift (Figs. 1c, 3b, and Supplementary Fig. 3I). Taken together, this indicates βArr1-3A is modulating the exchange kinetics of pre-existing agonist-bound conformations of the PIF motif – consistent with the proposed β2-adrenergic receptor activation mechanism^50^.

### BAM potentiates the exchange dynamics of the βArr-ternary complex

To test if exchange kinetics of the receptor’s conformational ensemble plays a general role in arrestin activation, we investigated ML314 ternary and ML314:NT8-13 quaternary complexes. The ML314:enNTS_1_ΔM4:βArr1-3A complex spectrum was qualitatively quite similar to NT8-13:enNTS_1_ΔM4:βArr1-3A with enhanced μs-ms exchange peripheral to the orthosteric pocket and slower ms-s motions near to the transducer interface (Fig. 3b, c). Yet, there were several key differences. M352^7.36^ state B remained visible at 2.3 molar equivalents βArr1-3A and even increased in intensity relative to ML314 alone (Fig. 3c); thus βArr1-3A stabilizes the ML314 bound enNTS_1_ΔM4 conformer, which we hypothesize reflects a detached N-terminus and tightly packed TM1/TM2/TM7 interface^22^. M244^5.45^ splits into at least five peaks including substates populated in the presence of ML314 and NT8-13:βArr1-3A (Fig. 3b–e). ML314 maintains M250^5.51^ in the furthest downfield position of any ligand with βArr1-3A perturbing it even further and simultaneously splitting it into an ensemble of at least three substates (Fig. 3c, f). As βArr1-3A also pushes M250^5.51^ downfield relative to NT8-13:enNTS_1_ΔM4, we hypothesize this chemical environment signifies a transducer-competent conformer (Fig. 3b). The simultaneous addition of ML314 and NT8-13 to enNTS_1_ΔM4:βArr1-3A collapses both M244^5.45^ and M250^5.51^ to a subset of resonances that more generally reflect a concatenation of the ML314:βArr1-3A and NT8-13:βArr1-3A ternary complexes (Fig. 3b–f).

### NTS_1:_Gα_iq_ conformational and kinetic ensemble is distinct from NTS_1:_βArr1-3A

**Fig. 4:**
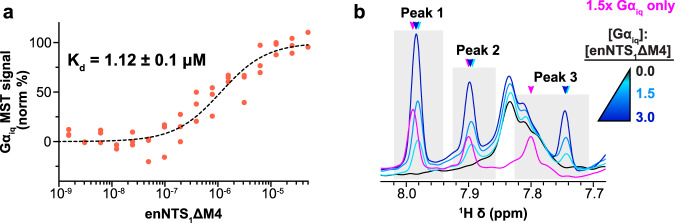
Measurement of Gα_iq_:enNTS_1_ΔM4 complex affinity. **a** Microscale thermophoresis (MST) measured the affinity of Gα_iq_ for NT8-13:enNTS_1_ΔM4 as 1.12 ± 0.1 μM (±SEM) using a single-site quadratic binding model. Data was collected as *n* = 3 biologically independent experiments in technical triplicates. Source data are provided as a Source Data file. **b** ^1^H 1D spectra of three unassigned Gα_iq_ resonances exhibiting enNTS_1_ΔM4-dependent chemical shift perturbations. Spectra were recorded at 600 MHz with receptor concentrations of 64 μM and variable Gα_iq_ concentrations: 0 μM (0×), 96 μM (1.5×), and 192 μM (3×).

NTS_1_ can uniquely couple to all major Gα protein subtypes (Gα_q/11_, Gα_i/o_, Gα_s_, and Gα_12/13_)^51^ with the strongest preference towards G_q_ activation. This reflects a higher affinity and nucleotide exchange rate for Gα_q_, at least compared to Gα_i_, primarily driven by the six C-terminal residues of the α5 helix^52^. Since Gα_q_ is inherently unstable^53,54^, we took advantage of the Gα_iq_ chimera originally used to demonstrate that coupling specificity can largely be reduced to the G protein C-terminus^52^. The enNTS_1_ΔM4:Gα_iq_ chimera complex affinity was 1.12 ± 0.1 μM, which corresponds to >98% receptor occupancy at a 2:1 transducer:receptor ratio and assuming a single-site ligand depletion binding model (Fig. 4a). NT8-13:enNTS_1_ΔM4:Gα_iq_ complex formation was further supported by concentration-dependent changes in 1D ^1^H and 2D ^1^H-^13^C HMQC spectra of [^13^C^ε^H_3_-methionine]-enNTS_1_ΔM4:Gα_iq_ (Fig. 4b and Supplementary Fig. 4) as well as a ^1^H-^15^N TROSY-HSQC spectrum of [*U*-^15^N,^13^C,^2^H]-[Gα_iq_:enNTS_1_ΔM4 (Supplementary Fig. 5a). All samples included apyrase to maximize complex affinity^24,55^, and tris-(2-carboxyehtyl)-phosphine (TCEP) to limit Gα_iq_ chimera self-association, which both had little to no observable effect on receptor resonances (Supplementary Fig. 5b). Two additional resonances, originating from unlabeled Gα_iq_ protein, are observed in the 2D ^1^H-^13^C HMQC spectrum but cannot be assigned to specific residues (Supplementary Fig. 6a).

**Fig. 5:**
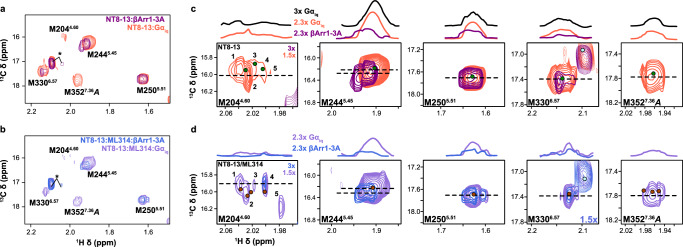
Comparison of Gα_iq_:enNTS_1_ΔM4 and βArr1-3A:enNTS_1_ΔM4 ternary complex NMR spectra. **a** Overlay of NT8-13:enNTS_1_ΔM4:βArr1-3A (purple) and NT8-13:enNTS_1_ΔM4:Gα_iq_ (tomato) ^1^H-^13^C HMQC spectra. Peaks marked with an asterisk represent natural abundance βArr1-3A and Gα_iq_ resonances (Supplementary Figs. 2b and 6a). **b** Overlay of NT8-13:ML314:enNTS_1_ΔM4:βArr1-3A (royal blue) and NT8-13:ML314:enNTS_1_ΔM4:Gα_iq_ (medium purple) ^1^H-^13^C HMQC spectra. Peaks marked with an asterisk represent natural abundance βArr1-3A and Gα_iq_ resonances. Extracted spectra regions of enNTS_1_ΔM4:Gα_iq_ and enNTS_1_ΔM4:βArr1-3A methionine resonances in the presence of (**c**) NT8-13 and (**d**) NT8-13:ML314. One dimensional ^1^H cross-sectional slices (corresponding to dashed lines in 2D spectra) shown on top. Dots denote the residue’s chemical shift position in spectra of the corresponding color with additional dots shown for ligand-only spectra (NT8-13:enNTS_1_ΔM4, forest green; NT8-13:ML314:enNTS_1_ΔM4, maroon). Gα_iq_ containing spectra were recorded at 600 MHz with receptor concentrations of 64 μM and βArr1-3A containing spectra with receptor concentration of 66 μM.

A comparison of the Gα_iq_ and βArr1-3A titration series is particularly revealing (Fig. 5a–d and Supplementary Figs. 3 and 4). M250^5.51^ and M330^6.57^ concomitantly split into multiple, overlapping resonances in both ternary complexes, but βArr1-3A qualitatively promotes these changes at a slightly lower relative concentration. It is reasonable to anticipate a subset of similarly behaving resonances considering the highly conserved receptor architecture observed across all transducer complex structures and presumably partially-overlapped allosteric coupling networks^23–26^. There are three striking differences between Gα_iq_ and βArr1-3A ternary complex spectra. First, M244^5.45^ remains a single unperturbed resonance in the presence of up to 3x molar excess of Gα_iq_. We do not observe any changes, apart from a subtle intensity reduction, suggesting that (i) NT8-13 alone induces a fully-active, G protein-competent M244^5.45^ conformation; (ii) the isolated Gα_iq_ subunit is insufficient to stabilize the fully-active state; or (iii) that M244^5.45^ does not play a role in Gα_iq_ coupling. Secondly, both transducers split M352^7.36^ state A into at least two resonances but βArr1-3A leads to exchange broadening at lower concentrations (Fig. 5a–d and Supplementary Figs. 3k and 4k). Finally, Gα_iq_ stabilizes three M204^4.60^ resonances while βArr1-3A broadens all peaks before selecting a single state at 3x molar equivalents (Fig. 5a–d and Supplementary Figs. 3g and 4g). A detailed comparison of M204^4.60^ is challenging due to the overall weak intensities and similar resonance patterns observed at sub-stoichiometric transducer concentrations (Fig. 5a–d and Supplementary Figs. 3g and 4g). Yet, taken together, these chemical shift and intensity changes suggest that Gα_iq_ and βArr1-3A differentially modulate the kinetics of enNTS_1_ΔM4 conformational ensembles near the connector region and orthosteric binding pocket.

### BAM stabilizes a distinct Gα_iq_ quaternary complex

Although multiple studies confirm that ML314 attenuates G protein activation and downstream signaling^20–22^, it’s unclear if this results from a reduction in NTS_1_-G protein complex affinity, nucleotide exchange rate, or unproductive complex conformation. Surprisingly, MST measurements reveal that ML314 only modestly reduces the affinity of Gα_iq_ for NT8-13:enNTS_1_ΔM4 to 1.76 ± 0.2 μM (Supplementary Fig. 6b). We collected a ML314:NT8-13:enNTS_1_ΔM4:Gα_iq_ spectrum for insight into the mechanism by which ML314 attenuates G protein activation. We observe differential effects across the receptor with residues adjacent to the orthosteric pocket appearing to adopt more βArr1-competent-like conformers and those near the transducer-binding interface continuing to populate Gα_iq_-competent conformers. For example, ML314 reduces the M352^7.36^ state A intensity as observed in βArr1 ternary and quaternary complexes (Fig. 5a–d and Supplementary Figs. 3 and 6). At the bottom of the orthosteric pocket, ML314 again pushes M204^4.60^ along a linear trajectory towards a state that is only observed in βArr1-3A ternary and quaternary complexes, we hypothesize this may affect the hydrogen-bond network^22^ that governs receptor activation (Supplementary Fig. 3g). M244^5.45^ shows no substantial difference, apart from a subtle reduction in peak intensity, between NT8-13:enNTS_1_ΔM4, ML314:NT8-13:enNTS_1_ΔM4 and NT8-13:enNTS_1_ΔM4:Gα_iq_ (Supplementary Fig. 6). Whereas residue M250^5.51^, the closest probe to the transducer interface, begins to reflect a concatenation of Gα_iq_ and βArr1-competent states (Fig. 5a–d and Supplementary Fig. 6). Perhaps the most dramatic change is observed in the M330^6.57^ multiplet pattern. While titration of either transducer initially splits M330^6.57^ in the ^1^H dimension, βArr1 ultimately stabilizes the downfield resonance (Supplementary Figs. 3 and 4).

## Discussion

Over the last two decades, extensive crystallographic and cryo-electron microscopy studies have laid a structural foundation for GPCR activation in terms of inactive, intermediate, and active-state models. Sophisticated spectroscopic and computational studies expanded the conformational landscape to include high energy intermediate states from the fs-ns fluctuation of bond angles and side chain rotamers^56^ to the ns-μs toggling of microswitches^36,50,57,58^, the μs-ms conformational exchange of secondary structure^35,59^, and the ms-s activation of transducers^59,60^. Site-selective NMR labeling strategies, which are sensitive to molecular motions over the picosecond to second timescale, have proven especially powerful for describing how ligands and transducers remodel the GPCR conformational landscape^27,61^. Here, we employed endogenous ^13^C^ε^H_3_-methionine probes located around the extracellular vestibule and near the connector region to expand our understanding of how ligands, allosteric modulators and transducers regulate NTS_1_ motions.

X-ray crystallography and MD studies suggest that ligand binding is communicated to the transducer interface through correlated motions near the connector or transmission region^50,62^. To further explore PIP2-mediated cooperativity between the orthosteric pocket (M330^6.57^) and connector region (M244^5.45^ and M250^5.51^), we measured the pairwise correlation of normalized peak intensities for each liganded state (Supplementary Fig. 7). As all spectra were collected using the same acquisition parameters on identically-prepared samples, the differential peak volumes should reflect individual or cumulative dynamics across the fast (ps-ns), intermediate (μs-ms), and slow (ms-s) NMR timescales. For example, ps-ns motions suggested by DFT analysis^22,63^ could alter peak intensities through transverse (T_2_) or longitudinal (T_1_) relaxation mechanisms^64^, although we hypothesize that the SOFAST-HMQC experiments employed dramatically reduce the possibility of differential T_1_ relaxation^65^. Slow exchange dynamics, such as peak splitting, were controlled for by summing the intensities across all resonances of a given residue; thus, we attribute differences to motions on the sub-microsecond timescale. Nonetheless, future experiments will be required to quantitate the T_1_, T_2_, and generalized order parameters for each methionine methyl group.

**Fig. 6:**
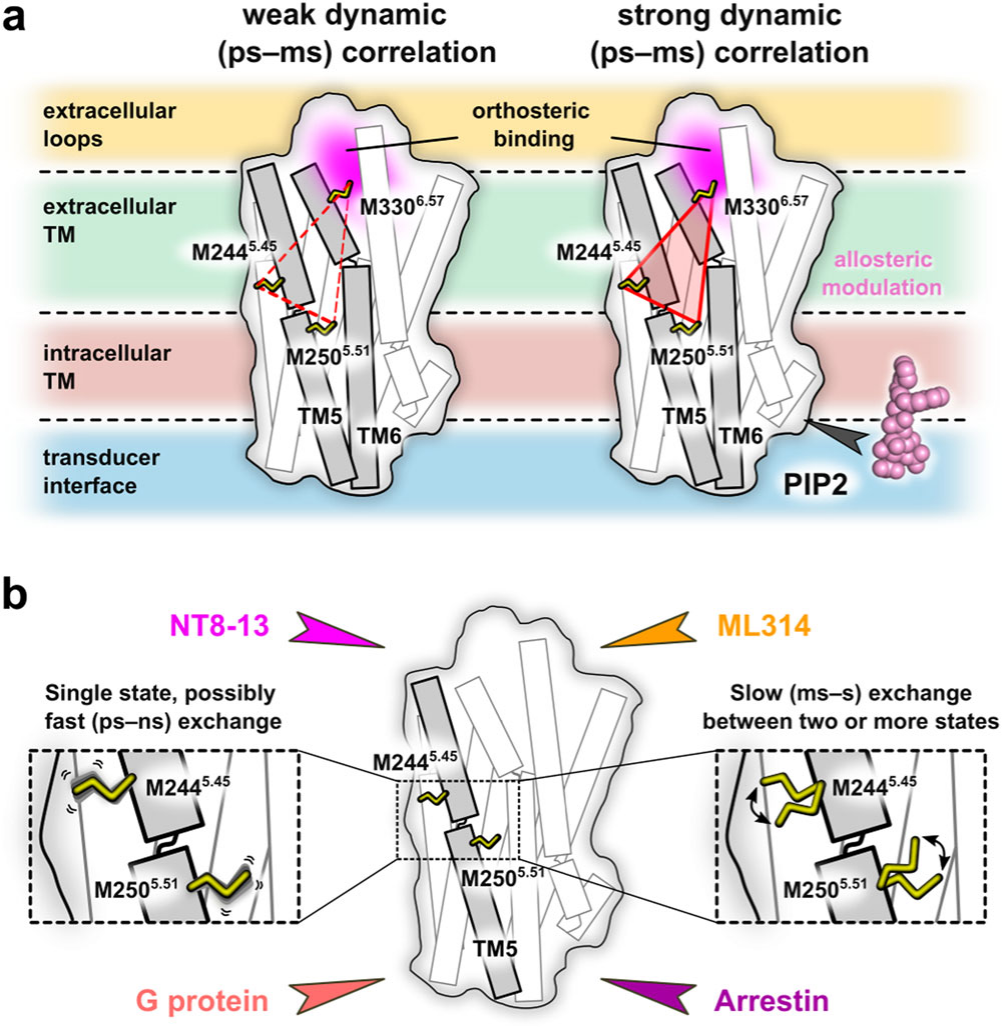
Ligands and transducers remodel the enNTS_1_ kinetic and/or thermodynamic ensemble. **a** The weak pair-wise correlation of peak intensities near the orthosteric pocket and connector region are strengthened by PIP2 to reveal several long-range allosteric communication pipelines. **b** β-arrestin-1 association slows the timescale of M244^4.45^ and M250^5.51^ conformational exchange whereas G protein coupling has little to no effect. Our NMR spectra qualitatively suggest the pre-existence of transducer-competent conformations in the agonist-bound state and that ML314, a β-arrestin biased allosteric modulator (BAM), fine-tunes exchange between those states.

This analysis relies on the assumption that residue pairs involved in the same allosteric network will exhibit a concerted response – reminiscent of chemical shift covariance analysis (CHESCA)^66^ and methionine chemical shift-based order parameter analysis^22,63^. Residues M244^5.45^ and M250^5.51^ which are located before and after P249^5.50^, respectively, inform on the dynamics across the TM5 kink; the effect is relatively consistent regardless of PIP2, although the better linear correlation suggests an improved dynamic scaling between those residues. Similar results are observed for the pairwise correlation of M244^5.45^ and M330^6.57^, hinting at a subtle allosteric effect throughout the extracellular vestibule. Lastly, we looked at the correlation between M250^5.51^ and M330^6.57^. In the absence of PIP2, the two residues are effectively uncoupled (*R*^2^ = 0.14) but lipid addition increases the *R*^2^ to 0.69 and the slope to 0.99. The modest pairwise peak intensity correlations (Supplementary Fig. 7) between the orthosteric pocket and connector region alludes to a long-range allosteric coupling (Fig. 6a) and potential mechanism for PIP2’s ability to stabilize active states by strengthening the pairwise peak intensity correlations.

Linking our observation to a specific enNTS_1_ΔM4:PIP2 interface is challenging. A hNTS_1_:βArr1-ΔCT cryo-EM structure^23^ and a native mass spectrometry (MS) approach^38^ both indicate PIP2 binds at the TM1/2/4 groove; however, the same MS study proposes an additional interface formed by TM1/7^38^. Binding is dominated by electrostatic interactions between the polyanionic phosphorylated inositol head group of PIP2 and basic residues (Arg and Lys) of the receptor^23,38,67,68^. In the case of enNTS_1_, directed evolution resulted in three mutations (N262^5.63^R, K263^5.64^R and H305^6.32^R) at the intracellular tips of TMs 5 and 6, which could elevate the relevance of this second site^39,40^. NT8-13:ML314:enNTS_1_ΔM4 was the only ligand complex to exhibit substantial chemical shift changes upon addition of PIP2 (Fig. 1e). Perhaps this reflects counteracting allosteric networks, or perhaps even direct binding competition, of a balanced (PIP2) and biased (ML314) ligand in the absence of transducer. The latter may also be inferred from the Apo-M244^5.45^ peak splitting observed on addition of PIP2 (Fig. 2d) resulting in a similar peak shape and intensity compared to ML314:enNTS_1_ΔM4 and ML314:enNTS_1_ΔM4:PIP2. However, we cannot exclude the possibility that spectral changes are unrelated to transducer-competent conformations.

Recent cryo-EM structures of hNTS_1_:βArr1 and hNTS_1_:Gα_i_βγ ternary complexes possess very high receptor structural similarity, which raises questions as to the role of conformational dynamics in functional selectivity. Although technically challenging, other studies have also begun to integrate kinetic information for a more complete description of conformational landscapes^69–71^. Both M244^5.45^ and, to a lesser extent, M250^5.51^ resonances split in response to βArr1 but not Gα_iq_ (Figs. 5c and 6b). This peak pattern is indicative of a chemical exchange process on the slow NMR timescale suggesting a reasonably high activation barrier between substates. Interestingly, the βArr-biased allosteric modulator ML314 alone (or in combination with NT8-13) also induces splitting of those resonances while PIP2 splits Apo-state M244^5.45^ analogous to M314, suggesting pre-selection of βArr-binding-competent states and offering a mechanistic basis for transducer-bias. Although speculative, we hypothesize that peak splitting in the ^1^H dimension may originate from local fluctuations of one or several aromatic rings^22,63^. Finally, it is interesting to note the continued presence of Gα_iq_ competent chemical shifts in the ML314:NT8-13:enNTS_1_ΔM4:Gα_iq_ spectrum (Fig. 4c–f). Similar studies using enNTS_1_^19^F-labeled at the cytoplasmic tip of TM6 (Q301C^6.28^)^33^ reveal that a peptide corresponding to the G_q_ α5-helix binds ML314:enNTS_1_ΔM4 complexes with high-affinity by stabilizing unique TM6 conformers. Taken together, we hypothesize that ML314, and its successor SBI-553^72,73^, may bias NTS_1_ pharmacology by stabilizing signaling incompetent G protein conformations, such as a non-canonical Gα subunit^24,25^ and/or α5-helix pose^74,75^. Our observation that βArr1 but not G protein selects a pre-existing enNTS_1_ conformation near the highly conserved PIF motif is consistent with a previous NMR study^33^ that utilized a ^19^F probe at the intracellular tip of TM6 of enNTS_1_, suggesting that this conformational selection mechanism extends from the PIF motif within the TM domain across the intracellular transducer binding region. The observed peak splitting of M244^5.45^ and M250^5.51^ is suggestive of conformational exchange at the ms-s timescale which agrees with the slow-exchange regime established for the TM6 movements of enNTS_1_ by Dixon *et al*. However, they observed enNTS_1_ TM6 adopts a new conformational state upon Gα_q_-peptide binding (induced-fit) which we can neither confirm nor exclude based on our current data.

Our model system employed several strategies to minimize the challenges to solution NMR studies of GPCR complexes such as multiple simultaneous binding partners, heterogenous phosphorylation patterns, high molecular weights, and inherent instability. We employed the pre-activated βArr-3A variant to control for uncertainty related to the number and position of phosphorylated residues in the receptor C-terminus. To minimize line-broadening side effects of slowly tumbling systems, we employed DDM detergent micelles and focused on only the Gα subunit that comprises nearly the entire complex interface. The Gα_iq_ chimera provides a robust scaffold to explore an otherwise unstable cognate receptor:G protein pair^53,54^. Thermostabilized enNTS_1_ permits extended data acquisition times that would otherwise be impossible; it binds ligands with similar affinity to rNTS_1_^76^, and couples directly to G protein and β-arrestin, although with reduced affinity. Future studies will explore reversion of thermostabilizing mutations to further recover wildtype signaling capabilities. Nonetheless, loss-of-function mutations are more common than gain-of-function phenotypes suggesting that the molecular mechanisms of enNTS_1_/transducer coupling represent native allosteric pipelines. A long-term aim is to couple quantitative sidechain motions with all atom molecular dynamics to map allosteric connection pathways. Accurate quantitation requires highly deuterated systems^28,77^ that can be achieved by elegant means^78–80^, but are most easily afforded by *E. coli* expression systems.

## Methods

### *E. coli* expression and purification of enNTS_1_ΔM4

^13^C^ε^H_3_-methionine labelled enNTS_1_ΔM4 (M204^4.60^/M208^4.64^/M244^5.45^/M250^5.51^/M330^6.57^/M352^7.36^) was expressed as a MBP-enNTS_1_ΔM4-muGFP fusion protein using the following protocol. 5 mL of a LB day-pre-culture containing 100 mg/L ampicillin and 1% (w/v) glucose were inoculated with a single colony of E. coli OverExpress C43(DE3) cells (Sigma-Aldrich) freshly transformed with pDS170-enNTS_1_. After 9 h (37 °C, 225 rpm) this pre-culture was centrifuged (1700×*g*, at RT, 5 min) and the pellet used to inoculate 250 mL of a defined medium pre-culture and grown overnight (1 L flask, 37 °C, 225 rpm). The defined medium consisted of an autoclaved basal salts solution (30 mM KH_2_PO_4_, 23 mM K_2_HPO_4_, 16 mM Na_2_HPO_4_, 17 mM NaCl, 37 mM NH_4_Cl, adjusted to pH 7.4 with NaOH) supplemented with sterile filtered trace metal stock solution (1% v/v)^81^, 2 mM MgSO_4_, 0.4% w/v glucose, 50 mg/L thiamine and 100 mg/L ampicillin. After 13 h the defined medium pre-culture reached an OD600 of ~2, was centrifuged and its pellet resuspended into 250 mL of fresh medium of which 20 mL was used to inoculate 1 L of the same defined medium per 2.8 L flask. Expression cultures were grown for 6.5 h (37 °C, 225 rpm) to an OD600 of 0.4. The flasks were then cooled on ice for 2 min at which point 50 mg/L ^13^CH_3_-methionine (Cambridge Stable Isotopes) was added along with 100 mg/L each of lysine, threonine, phenylalanine, and 50 mg/L each of leucine, isoleucine and valine. The expression cultures were placed into a 16 °C incubator for 15 min, then induced with 250 mM isopropyl β-D-1-thiogalactopyranoside (IPTG) and protein expression was carried out at 16 °C and 225 rpm for 12–16 h. For unlabeled expression of MBP-enNTS_1_ΔM4-muGFP, 5 mL of LB pre-culture was used to inoculate 500 mL 2YT medium containing 100 mg/L ampicillin and 0.2% (w/v) glucose in 2 L flasks. The flasks were incubated at 37 °C and 225 rpm until an OD600 of 0.4 was reached. The flasks were cooled on ice for 2 min, then induced with 250 μM IPTG. Protein expression was carried out at 16 °C and 225 rpm for 12–16 h. All expression cultures were harvested by centrifugation (5000×*g*, 4 °C, 15 min) and the combined pellets resuspended with 50 mL of wash buffer (25 mM HEPES, 100 mM NaCl, pH 8) per 1 L of expression culture and centrifuged again (3000×*g*, 4 °C, 15 min). The cell pellets were kept frozen at −80 °C until further use.

Cell pellets equivalent to 3–4 L culture were thawed on ice, resuspended in 50 mL of 100 mM HEPES, 400 mM NaCl, 20% glycerol, pH 8 with an EDTA free protease inhibitor tablet (Roche), 100 mg lysozyme and 10 mg DNAse. After rocking for 30 min at 4 °C the cells were sonicated on ice; mixed with 15 mL of DM solution (1.6 g n-decyl-β-D-maltopyranoside, Anatrace dissolved in 15 mL MilliQ water) and 15 mL of CHS/CHAPS solution (0.018 g cholesterol hemi succinate (CHS, Sigma) and 0.09 g 3-((3-cholamidopropyl) dimethylammonio)−1-propanesulfonate hydrate (CHAPS-hydrate, Sigma) dissolved in 15 mL MilliQ water). The solubilization mix was rocked for 2 h at 4 °C; centrifuged (12,000×*g*, 4 °C, 30 min) and the supernatant was filtered using a 45 μm syringe filter (Millipore), adjusted to 10 mM imidazole and mixed with 3 mL of Talon resin equilibrated in 25 mM HEPES, 300 mM NaCl, 10% glycerol, 0.15% DM, pH 8 and rocked for 1.5 h at 4 °C. The resin retaining the receptor was washed twice with 25 mL of 25 mM HEPES, 500 mM NaCl, 10% glycerol, 0.15% DM, 10 mM imidazole, 0.2 mM PMSF (phenylmethylsulfonyl fluoride), 8 mM ATP, 10 mM MgCl_2_, pH 8. Detergent exchange to DDM (n-dodecyl-β-D-maltopyranoside, Anagrade, Anatrace) was initiated by washing the resin twice with 25 mL of 25 mM HEPES, 100 mM NaCl, 10% Glycerol, 0.05% DDM, 0.2 mM PMSF, pH 8. The fusion protein was eluted with 15 mL of 25 mM HEPES, 100 mM NaCl, 10% glycerol, 0.05% DDM, 350 mM imidazole, 0.2 mM PMSF, pH 8. IMAC elutions were cleaved with HRV 3 C protease (produced in-house) prior to concentrating using an Amicon 30 kDa MWCO concentrator (Millipore) and dilution with ion exchange chromatography (IEX) loading buffer (20 mM HEPES pH 8.0, 10% Glycerol, 0.02% DDM) to obtain a combined NaCl/Imidazole/Na_2_SO_4_ concentration of less than 50 mM. The cleaved receptor solution was then loaded onto a 5 mL HiTrap SP HP column (GE Healthcare) using an Akta Start system (GE Healthcare) and washed with the same buffer until the signal remained stable. The column was then washed with four column volumes of IEX wash buffer (20 mM HEPES pH 7.4, 10% Glycerol, 63 mM NaCl, 0.02% DDM) after which a 1 mL Ni-NTA HisTrap column (GE Healthcare) was inserted after the HiTrap SP HP column and the system was washed with another 10 mL of IEX wash buffer containing 10 mM Imidazole. The cleaved receptor was then eluted with IEX elution buffer (20 mM HEPES pH 7.4, 10% Glycerol, 1 M NaCl, 0.03% DDM, 20 mM Imidazole) and the receptor containing fractions concentrated to ~400 μL for injection onto a S200 Increase SEC column (GE Healthcare) using a 500 μL loop and an Akta Pure System (GE Healthcare). The receptor containing fractions from SEC purification using SEC buffer (50 mM Potassium phosphate pH 7.4, 100 mM NaCl, 0.02% DDM) were then concentrated and buffer exchanged (for NMR experiments) using NMR buffer (50 mM Potassium phosphate pH 7.4, 100 mM NaCl in 100% D_2_O) to reduce the residual H_2_O concentration to <1%. Receptor samples were then aliquoted and stored at −80 °C until further use. enNTS_1_ΔM4 used in NMR experiments retains a C-terminal Avi-tag (which was used previously for capture in ligand-binding and thermostability assays) and the amino acid sequence is:

GPGSTSESDTAGPNSDLDVNTDIYSKVLVTAIYLALFVVGTVGNGVTLFTLARKKSLQSLQSRVDYYLGSLALSSLLILLFALPVDVYNFIWVHHPWAFGDAGCKGYYFLREACTYATALNVVSLSVERYLAICHPFKAKTLLSRSRTKKFISAIWLASALLSLPMLFTMGLQNLSGDGTHPGGLVCTPIVDTATLRVVIQLNTFMSFLFPMLVASILNTVIARRLTVLVHQAAEQARVSTVGTHNGLEHSTFNVTIEPGRVQALRRGVLVLRAVVIAFVVCWLPYHVRRLMFVYISDEQWTTALFDFYHYFYMLSNALVYVSAAINPILYNLVSANFRQVFLSTLASLSPGWRHRRKKRPTFSRKPNSVSSNHAFSTASGLNDIFEAQKIEWHEGSGLEVLFQ

### Expression and purification of βArr1-3A

The pET15 expression plasmid harboring the cysteine-free hβArr1 gene (where 6 cysteine residues were mutated to other amino acid types: C59V, C125S, C140L, C242V, C251V and C269S) was a kind gift from Ashish Manglik. In this plasmid the hβArr1-3A sequence was modified by mutating I386A, V387A, F388A (termed 3A mutant)^82^ via site-directed mutagenesis (using the forward and reverse primer 5′-GAG TTA GAT ACT AAT GAT GAT GAT GCG GCC GCG GAG GAC TTT GCA CGC CAG CG-3′ and 5′-CGC TGG CGT GCA AAG TCC TCC GCG GCC GCA TCA TCA TCA TTA GTA TCT AAC TC-3′, respectively). hβArr1-3A gene was preceded by a 6x His tag, HRV 3 C protease cleavage site and Protein C tag. This sequence was modified by inserting an additional HRV 3C protease cleavage site between the Protein C sequence and the hβArr1-3A gene to allow complete removal of N-terminal tags. Insertion of the additional HRV 3C protease cleavage site was done by site directed mutagenesis (using the forward and reverse primer 5′-CCT GAT TGA TGG CAA AGG CGG AAG TGG AGG ACT GGA AGT GCT GTT CCA GGG CCC GTC GGG CGA CAA GGG AAC ACG TGT CTT CAA G-3′ and 5′-CTT GAA GAC ACG TGT TCC CTT GTC GCC CGA CGG GCC CTG GAA CAG CAC TTC CAG TCC TCC ACT TCC GCC TTT GCC ATC AAT CAG G-3′, respectively). 5 mL of a LB day pre-culture containing 100 mg/L carbenicillin and 1% (w/v) glucose were inoculated with a single colony of *E. coli* BL21(DE3) cells (Lucigen, Middleton, WI) freshly transformed with the βArr1-3A expression plasmid. After 9 h (37 °C, 225 rpm) 10 μL of LB pre-culture were added to 50 mL of a Terrific Broth (TB) pre-culture containing 100 mg/L carbenicillin and 1% (w/v) glucose, and incubated overnight at (30 °C, 225 rpm). The next morning the 50 mL of TB pre-culture were added to shaker flasks containing 950 mL of the same medium and incubated (37 °C, 225 rpm) to reach an OD600 of 0.6 at which point the temperature was reduced to 20 °C and the culture flasks were incubated further until an OD600 of 1.0 was reached. The flasks were then cooled on ice for 5 min prior to induction with 0.4 mM IPTG. Protein expression was carried out at 20 °C and 225 rpm for 19 h. The cells were harvested by centrifugation (5000 × g, 4 °C, 15 min) and the combined pellets resuspended with wash buffer (25 mM HEPES, 100 mM NaCl, pH 8) and the washed cells were then pelleted by centrifugation (3000×*g*, 4 °C, 15 min) and stored at −80 °C. Thawed cells were resuspended in solubilization buffer (20 mM HEPES pH 8, 500 mM NaCl, 2 mM MgCl_2_, 15% glycerol, 1 Roche EDTA free Protease inhibitor tablet, 0.4 mM PMSF, 1 mg/mL Lysozyme, 1 μL/mL DNAse) and left stirring at 4 °C for 30 min prior to sonication on ice. Cell debris was removed by centrifugation (24,000×*g*, 4 °C, 45 min) and the supernatant filtered using a 45 μm syringe filter (Millipore). The filtrate was then incubated for 1 h rotating at 4 °C with 2 mL Ni-NTA resin (Thermo Fisher) per 1.5 L of expression culture. The resin was then washed with 15 mL wash buffer 1 (20 mM HEPES pH 8, 300 mM NaCl, 10% glycerol, 10 mM imidazole) followed by 12 mL wash buffer 2 (same as wash buffer 1 but containing 20 mM imidazole) and 12 mL wash buffer 3 (same as wash buffer 1 but containing 25 mM imidazole) per 1 mL of resin. βArr1-3A was eluted with ~10 mL of elution buffer (20 mM HEPES pH 7.5, 150 mM NaCl, 10% glycerol, 200 mM imidazole) per 1 mL of resin. The 6× His-tag was removed via His-tagged HRV 3 C protease (produced in-house) cleavage overnight rotating at 4 °C. The cleavage reaction was then concentrated using an Amicon 30 kDa MWCO centrifugal concentrator (Millipore) and diluted with 20 mM HEPES pH 7.5 to obtain a combined NaCl/Imidazole/Na_2_SO_4_ concentration of less than 50 mM. The solution was again filtered using a 45 μm syringe filter (Millipore) prior to loading onto a 5 mL HiTrap Q IEX column (GE Healthcare) equilibrated with 20 mM HEPES pH 7.5 followed by a wash step with the same buffer until a conductivity of 5 mS/cm was reached. The column was then further washed with IEX wash buffer (20 mM HEPES pH 7.5, 50 mM NaCl) until the A280 signal stabilized. The column was eluted using a 25 min gradient stretching from 50 mM to 500 mM NaCl. The βArr1-3A containing fractions were then pooled and concentrated to ~750 μL using an Amicon 30 kDa MWCO centrifugal concentrator (Millipore) prior to injection onto a HiLoad 16/600 S200pg SEC column (GE Healthcare) equilibrated with SEC buffer (20 mM HEPES pH 6.8, 150 mM NaCl) using a 1 mL loop. The βArr1-3A containing SEC fractions were pooled, concentrated to 296 μM and aliquots stored at −80˚C until further use. The amino acid sequence of βArr1-3A used in NMR experiments is:

GPSGDKGTRVFKKASPNGKLTVYLGKRDFVDHIDLVDPVDGVVLVDPEYLKERRVYVTLTVAFRYGREDLDVLGLTFRKDLFVANVQSFPPAPEDKKPLTRLQERLIKKLGEHAYPFTFEIPPNLPSSVTLQPGPEDTGKALGVDYEVKAFVAENLEEKIHKRNSVRLVIRKVQYAPERPGPQPTAETTRQFLMSDKPLHLEASLDKEIYYHGEPISVNVHVTNNTNKTVKKIKISVRQYADIVLFNTAQYKVPVAMEEADDTVAPSSTFSKVYTLTPFLANNREKRGLALDGKLKHEDTNLASSTLLREGANREILGIIVSYKVKVKLVVSRGGLLGDLASSDVAVELPFTLMHPKPKEEPPHREVPENETPVDTNLIELDTNDDDAAAEDFARQRLKGMKDDKEEEEDGTGSPQLNNR

[*U*-^15^N,^13^C,^2^H]-βArr1-3A was produced using the above construct with a modified expression protocol. 5 mL of a LB day pre-culture containing 100 mg/L carbenicillin and 1% (w/v) glucose were inoculated with a single colony of *E. coli* BL21(DE3) cells (Lucigen, Middleton, WI) freshly transformed with the βArr1-3A expression plasmid. After 9 h (37 °C, 225 rpm), 250 μL of the LB pre-culture was centrifuged at 5000×*g*, resuspended in 5 mL LB/D_2_O containing 100 mg/L carbenicillin and 1% (w/v) glucose, and incubated overnight at (30 °C, 225 rpm). The next morning, 250 μL of the LB/D_2_O pre-culture was centrifuged at 5000×*g*, resuspended in 5 mL M9/D_2_O, and incubated (37 °C, 225 rpm). 1 L M9/D_2_O was prepared from: 1 L (99.9% ^2^H) D_2_O, 13.6 g Na_2_HPO_4_, 6 g KH_2_PO_4_, 1 g NaCl, 0.07 g CaCl_2_, 0.5 g MgSO_4_, 0.05 g carbenicillin, 1.5 g ^15^N_4_Cl, 3 g d7,^13^C-glucose, trace metal stock, and vitamin stock. After 9 h, the entire 5 mL M9/D_2_O pre-culture was added to 90 mL of a M9/D_2_O pre-culture and incubated overnight (30 °C, 225 rpm). The next morning, the 100 mL M9/D_2_O pre-culture was added to shaker flasks containing 900 mL of the same medium and incubated (37 °C, 225 rpm) to reach an OD600 of 0.8 at which point the temperature was reduced to 20 °C and the culture was incubated further until an OD600 of 1.0 was reached. The flasks were then cooled on ice for 5 min prior to induction with 0.4 mM IPTG. Protein expression was carried out at 20 °C and 225 rpm for 19 h. The cells were harvested by centrifugation (5000×*g*, 4 °C, 15 min) and the combined pellets resuspended with wash buffer (25 mM HEPES, 100 mM NaCl, pH 8) and the washed cells were then pelleted by centrifugation (3000×*g*, 4 °C, 15 min) and stored at −80 °C. Purification followed the same strategy as outlined above for unlabeled βArr1-3A.

For MST experiments, unlabeled βArr1-3A was expressed and purified as above, but the N-terminal 6xHis tag was left intact yielding the amino acid sequence:

MGSSHHHHHHLEVLFQGPGGEDQVDPRLIDGKGGSGGLEVLFQGPSGDKGTRVFKKASPNGKLTVYLGKRDFVDHIDLVDPVDGVVLVDPEYLKERRVYVTLTVAFRYGREDLDVLGLTFRKDLFVANVQSFPPAPEDKKPLTRLQERLIKKLGEHAYPFTFEIPPNLPSSVTLQPGPEDTGKALGVDYEVKAFVAENLEEKIHKRNSVRLVIRKVQYAPERPGPQPTAETTRQFLMSDKPLHLEASLDKEIYYHGEPISVNVHVTNNTNKTVKKIKISVRQYADIVLFNTAQYKVPVAMEEADDTVAPSSTFSKVYTLTPFLANNREKRGLALDGKLKHEDTNLASSTLLREGANREILGIIVSYKVKVKLVVSRGGLLGDLASSDVAVELPFTLMHPKPKEEPPHREVPENETPVDTNLIELDTNDDDAAAEDFARQRLKGMKDDKEEEEDGTGSPQLNNR

### Expression and purification of Gα_iq_

The codon optimized gene for the Gα_iq_ chimera^52^ was purchased from GenScript (Piscataway, NJ) in a puc57 vector and subcloned into a pIQ expression vector with an open reading frame encoding an N-terminal 6xHis tag followed by a NNNNNNNNNNG linker, a MBP sequence and a HRV 3 C protease cleavage site (LEVLFQGP). The Gα_iq_ gene was amplified via PCR using the forward and reverse primer 5′-CAT CAT GGA TCC GGT TGC ACC CTG TCT GCG GAA GAC-3′ and 5′-CAG CTA TGA CCA TGA TTA CGC-3′, respectively. Subcloning was performed via the BamHI and HindIII restriction sites. 50 mL of a LB day pre-culture containing 100 mg/L carbenicillin and 1% (w/v) glucose were inoculated with a single colony of *E. coli* BL21(DE3) cells (Lucigen, Middleton, WI) freshly transformed with the Gα_iq_ expression plasmid. After 9 h (37 °C, 225 rpm) 20 mL of LB pre-culture were centrifuged (3000×*g*, RT, 5 min) and the resuspended pellet was used to inoculate 1 L of 2xYT medium containing 100 mg/L carbenicillin and 0.2% (w/v) glucose. The cultures were incubated (37 °C, 225 rpm) to reach an OD600 of 0.7. The flasks were then cooled on ice for 5 min prior to induction with 1 mM IPTG. Protein expression was carried out at 25 °C and 225 rpm for 16 h. The cells were harvested by centrifugation (5000×*g*, 4 °C, 15 min) and the combined pellets resuspended with wash buffer (25 mM HEPES, 100 mM NaCl, pH 8) and the washed cells were then pelleted by centrifugation (3000×*g*, 4 °C, 15 min) and stored at −80 °C. Gα_iq_ was purified following a protocol for purification of miniG proteins^83^. A cell pellet from 2 L of *E. coli* culture was thawed on ice and resuspended in buffer A (40 mM HEPES pH 7.5, 100 mM NaCl, 10 mM imidazole, 10% v/v glycerol, 5 mM MgCl_2_, 50 μM GDP) to a volume of 50 mL. A protease inhibitor tablet (Roche), 1 mM PMSF, 5 U DNAse I, 25 mg lysozyme, and 100 μM DTT were added, and the cell suspension was stirred at medium speed at 4 °C for 30 min. The cells were then lysed by sonication on ice for 7 min, with pulses of 2 s on/4 s off, at an amplitude of 70% and the lysate centrifuged at 20,000×*g* for 45 min at 4 °C. The supernatant was filtered using a 45 μm syringe filter (Millipore) prior to loading onto a 5 mL HisTrap Fast Flow column (GE Healthcare), pre-equilibrated with ice-cold buffer A. The column was washed with 10 column volumes of ice-cold buffer B (20 mM HEPES pH 7.5, 500 mM NaCl, 40 mM imidazole, 10% v/v glycerol, 1 mM MgCl_2_, 50 μM GDP) and eluted with ~5 column volumes of ice-cold buffer C (20 mM HEPES pH 7.5, 100 mM NaCl, 500 mM imidazole, 10% v/v glycerol, 1 mM MgCl_2_, 50 μM GDP). The elute was complemented with 1 mM DTT, 100 mM Na_2_SO_4_, and His-tagged HRV 3 C protease (produced in-house) at a 3 C:Gα_iq_ ratio of 1:20 w/w, transferred to a 10 kDa cut-off dialysis tube (Thermo Fisher), and dialyzed overnight at 4 °C against 1 L of buffer D (20 mM HEPES pH 7.5, 100 mM NaCl, 10% v/v glycerol, 1 mM MgCl_2_, 10 μM GDP). The next day imidazole was added to a concentration of 20 mM and the solution was incubated with 4 mL of Ni-NTA resin (Thermo Fisher) pre-equilibrated with buffer D on a turning wheel for 30 min at 4 °C. The suspension was then poured onto 1 mL of fresh Ni-NTA resin (pre-equilibrated with buffer D) in a disposable plastic column and the flow-through was eluted by gravity. The column was washed with 5 mL of buffer D. The wash was combined with the flow-through and concentrated to ~1.6 mL using an Amicon 10 kDa MWCO centrifugal concentrator (Millipore) prior to loading onto a HiLoad 16/600 S200pg SEC column (GE Healthcare) pre-equilibrated with buffer E (10 mM HEPES pH 7.5, 100 mM NaCl, 10% v/v glycerol, 1 mM MgCl_2_, 1 μM GDP, 100 μM TCEP). The Gα_iq_ peak fractions were pooled and concentrated to ~0.5 mL followed by centrifugation at 16000×*g* for 5 min to remove aggregates. The solution containing 721 μM Gα_iq_ at a purity of >95% (as determined by SDS-Page) was flash-frozen in aliquots and stored at stored at −80 °C until further use. The amino acid sequence of Gα_iq_ used in NMR experiments is:

GPGSGCTLSAEDKAAVERSKMIDRNLREDGEKAAREVKLLLLGAGESGKSTIVKQMKIIHEAGYSEEECKQYKAVVYSNTIQSIIAIIRAMGRLKIDFGDSARADDARQLFVLAGAAEEGFMTAELAGVIKRLWKDSGVQACFNRSREYQLNDSAAYYLNDLDRIAQPNYIPTQQDVLRTRVKTTGIVETHFTFKDLHFKMFDVGGQRSERKKWIHCFEGVTAIIFCVALSDYDLVLAEDEEMNRMHESMKLFDSICNNKWFTDTSIILFLNKKDLFEEKIKKSPLTICYPEYAGSNTYEEAAAYIQCQFEDLNKRKDTKEIYTHFTCATDTKNVQFVFDAVTDVIIKNNLKEYNLV

[*U*-^15^N,^13^C,^2^H]-Gα_iq_ was produced using the above construct with a modified expression protocol. 5 mL of a LB day pre-culture containing 100 mg/L carbenicillin and 1% (w/v) glucose were inoculated with a single colony of *E. coli* BL21(DE3) cells (Lucigen, Middleton, WI) freshly transformed with the Gα_iq_ expression plasmid. After 9 h (37 °C, 225 rpm), 250 μL of the LB pre-culture was centrifuged at 5000×*g*, resuspended in 5 mL LB/D_2_O containing 100 mg/L carbenicillin and 1% (w/v) glucose, and incubated overnight at (30 °C, 225 rpm). The next morning, 250 μL of the LB/D_2_O pre-culture was centrifuged at 5000×*g*, resuspended in 5 mL M9/D_2_O, and incubated (37 °C, 225 rpm). After 9 h, the entire 5 mL M9/D_2_O pre-culture was added to 90 mL of a M9/D_2_O pre-culture and incubated overnight (30 °C, 225 rpm). The next morning, the 100 mL M9/D_2_O pre-culture was added to shaker flasks containing 900 mL of the same medium and incubated (37 °C, 225 rpm) to reach an OD600 of 0.8 at which point the temperature was reduced to 25 °C and the culture was incubated further until an OD600 of 1.0 was reached. The flasks were then cooled on ice for 5 min prior to induction with 1 mM IPTG. Protein expression was carried out at 25 °C and 225 rpm for 20 h. The cells were harvested by centrifugation (5000×*g*, 4 °C, 15 min) and stored at −80 °C. Purification followed the same strategy as outlined above for unlabeled Gα_iq_.

A second Gα_iq_ construct was cloned for MST experiments from the above template. The MBP and 3 C cleavage site were removed via restriction free cloning (using the forward and reverse primers 5′-GAG AGG ATC GCA TCA CCA TCA CCA TCA CGG ATC TGG ATC CGG TTG CAC CCT GTC TGC GGA AGA C-3′ and 5′-GTC TTC CGC AGA CAG GGT GCA ACC GGA TCC AGA TCC GTG ATG GTG ATG GTG ATG CGA TCC TCT C-3′, respectively) to place the 6xHis tag in closer proximity to the protein core. This construct was purified as above except for the 3C cleavage step to yield the amino acid sequence:

MRGSHHHHHHGSGSGCTLSAEDKAAVERSKMIDRNLREDGEKAAREVKLLLLGAGESGKSTIVKQMKIIHEAGYSEEECKQYKAVVYSNTIQSIIAIIRAMGRLKIDFGDSARADDARQLFVLAGAAEEGFMTAELAGVIKRLWKDSGVQACFNRSREYQLNDSAAYYLNDLDRIAQPNYIPTQQDVLRTRVKTTGIVETHFTFKDLHFKMFDVGGQRSERKKWIHCFEGVTAIIFCVALSDYDLVLAEDEEMNRMHESMKLFDSICNNKWFTDTSIILFLNKKDLFEEKIKKSPLTICYPEYAGSNTYEEAAAYIQCQFEDLNKRKDTKEIYTHFTCATDTKNVQFVFDAVTDVIIKNNLKEYNLV

### Microscale thermophoresis

βArr1-3A and Gα_iq_ chimera constructs containing N-terminal 6x His-tags were labeled with the His-Tag Labeling Kit RED-tris-NTA 2nd Generation (NanoTemper). A constant concentration of either RED-βArr1-3A or RED-Gα_iq_ chimera was incubated with varying concentrations of NT8-13-bound enNTS_1_ΔM4 before being loaded into NanoTemper premium capillaries and assayed via a NanoTemper Monolith NT MST instrument. βArr1-3A experiments consisted of 16 samples in 50 mM Potassium phosphate pH 7.4, 100 mM NaCl containing 25 nM RED-βArr1-3A, 40 µM NT8-13, 40 µM C8-PIP2, 0.03% DDM and a 1:2 dilution series of enNTS_1_ΔM4 from 20 μM to 0.61 nM. Gα_iq_ chimera experiments consisted of 16 samples in 50 mM Potassium phosphate pH 7.4, 100 mM NaCl containing 25 nM RED-Gα_iq_ chimera, 100 µM NT8-13, 100 µM C8-PIP2, 0.03% DDM, 2 mM MgCl_2_, 10 μM GDP, 0.2 units of apyrase and a 1:2 dilution series of enNTS_1_ΔM4 from 50 μM to 1.53 nM. Data was collected using 80% LED and 80% MST power in experimental triplicate and at least three technical repeats. Raw data was fit to a Hill function to establish upper and lower asymptote values. These values were then used to normalize the data and subsequently fit to a quadratic binding function using Sigma Plot v15 (Systat Software Inc.).

### NMR spectroscopy

NMR spectra were collected on 600 MHz Bruker Avance Neo spectrometers equipped with triple resonance cryoprobes and operated with Topspin v3.6.2. 2D ^1^H-^13^C SOFAST-HMQC spectra^65^ were recorded with 25% non-uniform sampling (NUS) at 298 K with a ^1^H spectral width of 12 ppm (1024 data points in t_2_) and a ^13^C spectral width of 25 ppm (128 data points in t_1_), relaxation delays of 450 ms, and 2048 scans per t1 data point resulting in acquisition times of 10 h per spectrum. A 2.25 ms PC9 120 degree ^1^H pulse^84^ was applied for excitation and a 1 ms r-SNOB shaped 180 degree ^1^H pulse^85^ was used for refocusing. The ^13^C carrier frequency was positioned at 17 ppm, and the ^1^H at 4.7 ppm, while band selective ^1^H pulses were centered at 1.8 ppm. 1D ^1^H spectra were recorded at 298 K with a spectral width of 13.7 ppm (2048 data points), a relaxation delay of 1 s, and 128 scans. ^15^N-TROSY-HSQC spectra were recorded at 298 K with 50% NUS using a Poisson gap schedule^86^, a ^1^H spectral width of 12 ppm (2048 data points in t_2_), a ^15^N spectral width of 40 ppm (128 data points in t_1_), relaxation delays of 1 s, and 128 scans per t_1_ data point resulting in acquisition times of ~9 h per spectrum.

The [*U*-^15^N,^13^C,^2^H]-βArr1-3A sample was prepared to a volume of 160 μL NMR buffer in 3 mm tubes (Wilmad), containing 20 μM DSS and 0.05% Na_2_N_3_. NT8-13 was added to a final concentration of 500 μM. PIP2 was added to a final concentration of 130 μM (~1.5x molar equivalents of the final receptor concentration). Unlabeled enNTS_1_ΔM4 was buffer exchanged three times with NMR buffer (to >99%) prior to combining with the transducer. Following each addition of unlabeled enNTS_1_ΔM4, the samples were incubated for 1 h at room temperature prior to starting experiments.

The [*U*-^15^N,^13^C,^2^H]-Gα_iq_ sample was prepared to a volume of 160 μL NMR buffer in 3 mm tubes (Wilmad) containing 20 μM DSS, 0.05% Na_2_N_3_, 2 mM MgCl_2_, 100 μM TCEP and 0.25 units apyrase. NT8-13 was added to a final concentration of 500 μM. PIP2 was added to a final concentration of 130 μM (~1.5x molar equivalents of final receptor concentration). Unlabeled enNTS_1_ΔM4 was buffer exchanged three times with NMR buffer (to >99%) prior to combining with the transducer. Following each addition of unlabeled enNTS_1_ΔM4, the samples were incubated for 1 h at room temperature prior to starting experiments.

[^13^C^ε^H_3_-methionine]-enNTS_1_ΔM4 samples were prepared to volumes of 160 μL in 3 mm tubes (Wilmad), containing 20 μM DSS and 0.05% Na_2_N_3_. Ligands were added to a final concentration of 500 μM. NT8-13 (5–10 mM) stock solutions were prepared in 100% D_2_O and ML314 (20 mM) in 100% DMSO-d6. PIP2 was added to a final concentration of 130 μM (~2× molar equivalents of receptor). βArr1-3A and Gα_iq_ aliquots of 0.3–3x molar equivalents of receptor were buffer exchanged three times with NMR buffer (to >99%) prior to combining with the receptor. βArr1-3A containing samples were incubated for 1 h at room temperature prior to starting experiments. Gα_iq_ containing samples were supplemented with 2 mM MgCl_2_, 100 μM TCEP, and 10 μM GDP and incubated for 1 h at room temperature prior to adding 0.25 units of Apyrase (NEB) and incubation for a further 1 h at room temperature.

All [^13^C^ε^H_3_-methionine]-enNTS_1_ΔM4 spectra were referenced against internal DSS, reconstructed with compressed sensing using qMDD^87^, and processed using NMRPipe^88^ where data were multiplied by cosinebells and zero-filled once in each dimension. All 2D ^1^H-^13^C SOFAST-HMQC spectra used in this study are reproduced together in Fig. S8.

Both [*U*-^15^N,^13^C,^2^H]-βArr1-3A and [*U*-^15^N,^13^C,^2^H]-Gα_iq_ spectra were referenced against internal DSS, reconstructed with iterative soft thresholding (IST)^86^, and processed using NMRPipe^88^ where data were multiplied by cosinebells and zero-filled once in each dimension.

All spectra were analyzed in NMRFAM Sparky v1.47 (Goddard, T.D. and Kneller, D.G., University of California, San Francisco). Peak integrals were normalized and plotted using GraphPad Prism v9.5.1 (GraphPad Software).

^13^C-SOFAST-HMQC spectra of Apo-state, NT8-13-, ML314-, and NT8-13 & ML314-bound enNTS_1_ΔM4 without PIP2 were generated in a previous study^22^.

### Reporting summary

Further information on research design is available in the Nature Portfolio Reporting Summary linked to this article.

## Supplementary information

Supplementary Information

Reporting Summary

## Data Availability

Source data used for graphs are provided with this paper. The chemical shift assignments of ^13^C-SOFAST-HMQC spectra generated in this study have been deposited in the Biological Magnetic Resonance Bank (BMRB) under accession codes 51908 (PIP2:Apo-state-enNTS_1_ΔM4), 51909 (PIP2:NT8-13:enNTS_1_ΔM), 51910 (PIP2:ML314:enNTS_1_ΔM4), 51911 (PIP2:NT8-13:ML314:enNTS_1_ΔM4), 51914 (NT8-13:enNTS_1_ΔM4:βArr1-3A), 51915 (PIP2:NT8-13:enNTS_1_ΔM4:βArr1-3A), 51916 (PIP2:ML314:enNTS_1_ΔM4:βArr1-3A), 51917 (PIP2:NT8-13:ML314:enNTS_1_ΔM4:βArr1-3A), 51921 (PIP2:NT8-13:enNTS_1_ΔM4:Gα_iq_), and 51927 (PIP2:NT8-13:ML314:enNTS_1_ΔM4:Gα_iq_). ^13^C-SOFAST-HMQC spectra of Apo-state, NT8-13-, ML314-, and NT8-13 & ML314-bound enNTS_1_ΔM4 were generated in a previous study^22^ and the chemical shift assignments were deposited in the BMRB under accession codes 51728 (Apo-state enNTS_1_ΔM4), 51735 (NT8-13:enNTS_1_ΔM), 51737 (ML314:enNTS_1_ΔM4), 51738 (NT8-13:ML314:enNTS_1_ΔM4). The NMR spectra generated during and/or analyzed during the current study are available from the corresponding author on reasonable request. PDB files referenced in this manuscript are available from the RCSB Protein Data Bank: 4BWB (HTGH4-ΔIC3:NT8-13), 6YVR (NTSR1-H4X:SR142948A, and 6Z4Q (NTSR1-H4X:NT8-13). Source data are provided with this paper.

## Source data

Source Data
