# Supplementary material for: Stabilization of pre-existing neurotensin receptor conformational states by β-arrestin-1 and the biased allosteric modulator ML314
Source: Nat Commun. 2023 Jun 7;14:3328. doi: 10.1038/s41467-023-38894-8 (PMC10247727; doi:10.1038/s41467-023-38894-8)
Supplement: Supplementary file 1 — Supplementary Information [file 41467_2023_38894_MOESM1_ESM.pdf]

Supplemental Information

Stabilization of pre-existing neurotensin receptor conformational states by β-arrestin-1 and the biased allosteric modulator ML314

Fabian Bumbak[1,2,*], James B. Bower[1,†], Skylar C. Zemmer[1,†], Asuka Inoue[3], Miquel Pons[4], Juan Carlos Paniagua[5], Fei Yan[6], James Ford[7], Hongwei Wu[7,8], Scott A. Robson[1], Ross A. D. Bathgate[9], Daniel J. Scott[9], Paul R. Gooley[6], and Joshua J. Ziarek[1,*]

[1]Department of Molecular and Cellular Biochemistry, Indiana University, Bloomington, Indiana, USA 47405
[2]Present address: ARC Centre for Cryo-electron Microscopy of Membrane Proteins and Drug Discovery Biology, Monash Institute of Pharmaceutical Sciences, Monash University, Parkville, Victoria 3052, Australia
[3]Graduate School of Pharmaceutical Sciences, Tohoku University, Sendai, Miyagi, Japan 980-8578
[4]Biomolecular NMR laboratory. Department of Inorganic and Organic Chemistry. Universitat de Barcelona (UB). 08028-Barcelona, Spain
[5]Department of Materials Science and Physical Chemistry & Institute of Theoretical and Computational Chemistry (IQTCUB). Universitat de Barcelona (UB). 08028-Barcelona, Spain
[6]Department of Biochemistry and Pharmacology, Bio21 Molecular Science and Biotechnology Institute, University of Melbourne, Parkville, Victoria 3010, Australia
[7]Department of Chemistry, Indiana University, Bloomington, Indiana 47405-7102, USA
[8]Present address: School of Chemistry & Biochemistry, Georgia Institute of Technology, Atlanta, Georgia 30332, USA
[9]The Florey Institute of Neuroscience and Mental Health and Department of Biochemistry and Pharmacology, The University of Melbourne, Parkville, Victoria 3010, Australia

[†]These authors contributed equally
*Corresponding authors: fabian.bumbak@monash.edu (FB) and jjziarek@gmail.com (JJZ)

Keywords: G protein-coupled receptors (GPCRs), NMR, allosteric modulator, conformational selection, Phosphatidylinositol-4,5-bisphosphate (PIP2), PIF motif, methionine

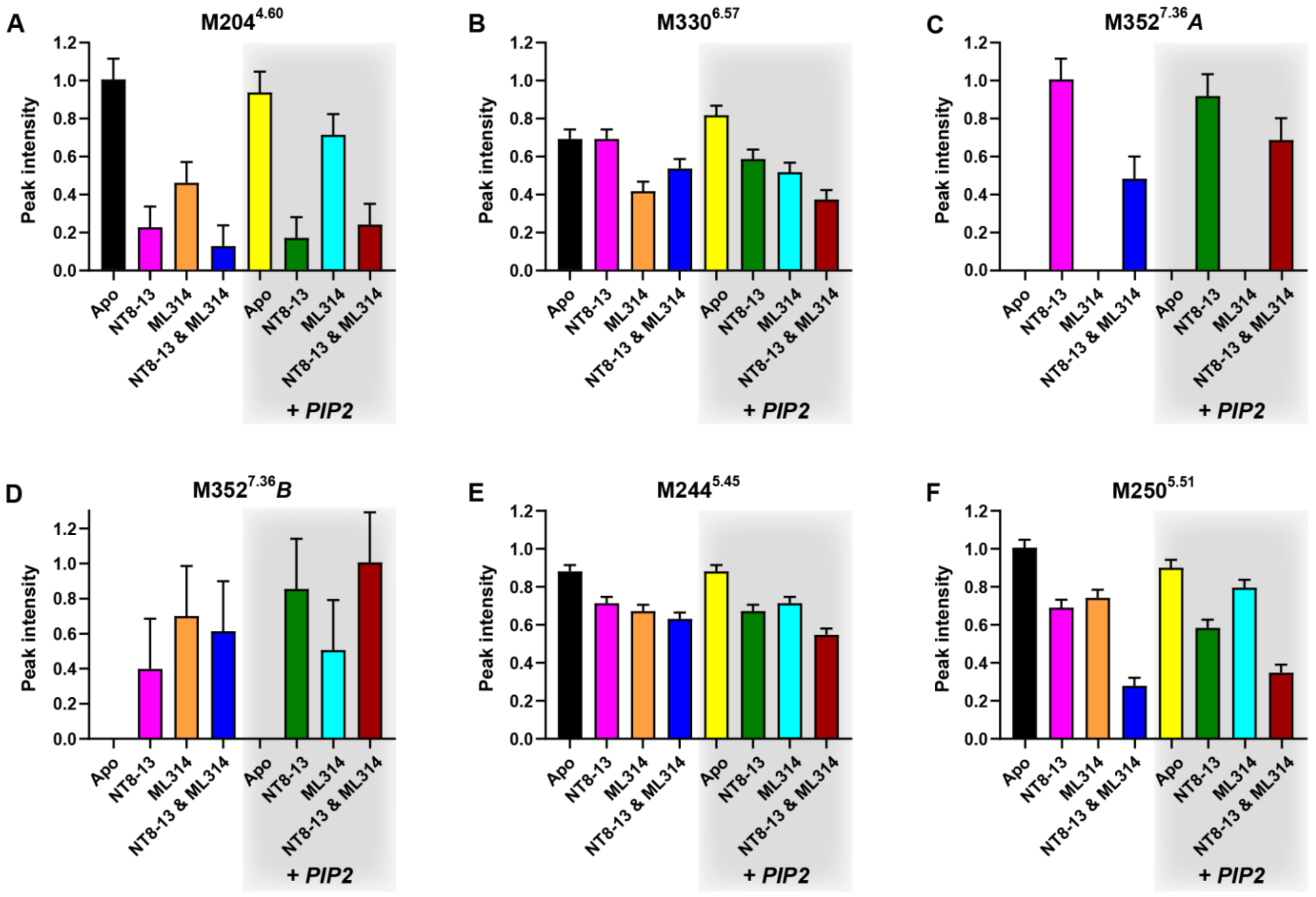

Supplementary Figure 1. Effect of ligand and PIP2 combinations on enNTS$_1$ΔM4 $^{13}C^{\varepsilon}H_3$-methionine peak volumes. $^{13}C^{\varepsilon}H_3$-methionine peak volumes of residues M204$^{4.60}$ (A), M330$^{6.57}$ (B), M352$^{7.36}$A (C), M352$^{7.36}$B (D), M244$^{5.45}$ (E), and M250$^{5.51}$ (F). Peak volumes were determined by manual integration using Sparky**.** For each residue, peak volumes were normalized to the largest valued condition. Bar colors correspond to the color scheme used for HMQC spectra. M204$^{4.60}$ intensities are the sum of all split resonances. Intensities for NT8-13 and NT8-13 & PIP2 conditions are the mean intensities from two independent samples. Error bars represent the standard deviation (SD) of peak volumes averaged for all 6 resonances in the NT8-13 and NT8-13 & PIP2 duplicate experiments. Source data are provided as a Source Data file.

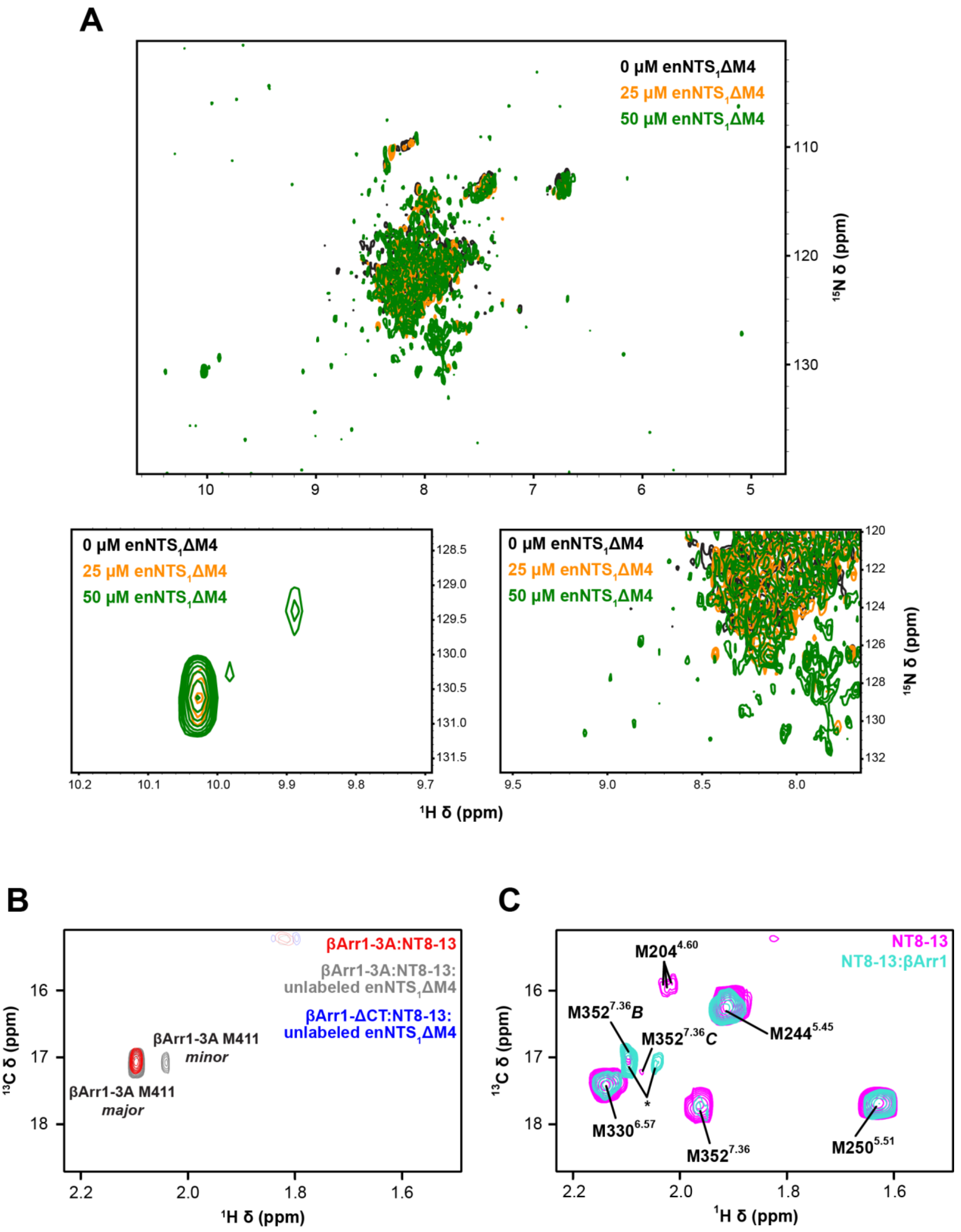

Supplementary Figure 2. enNTS$_1$ΔM4 interacts with βArr1-3A in the presence of PIP2. A) ^{1}H-^{15}N TROSY-HSQC spectra overlay of 50 µM [*U*-^{15}N,^{13}C,^{2}H]-βArr1-3A in the presence of 0 (black), 25 (orange) and 50 µM (green) unlabelled-enNTS$_1$ΔM4. All spectra were recorded at 600 MHz. The poor initial spectral quality likely reflects the inherent dynamics of pre-activated βArr1-3A. Nonetheless, chemical shift perturbations and increasing peak intensities are observed for a subset of resonances consistent with a specific interaction despite the substantial increase in rotational correlation time and sub-optimal TROSY field-strength. B) Comparison of 87 µM βArr1-3A (red), 165 µM βArr1-3A + 55 µM unlabelled-enNTS$_1$ΔM4 (grey), and 150 µM βArr1-ΔCT + 65 µM unlabelled-enNTS$_1$ΔM4 (blue) ^{1}H-^{13}C HMQC spectra. All spectra were collected in the presence of NT8-13 and PIP2 in DDM micelles. The two βArr1 resonances were both assigned to M411 because of their absence in the βArr1-ΔCT spectrum (blue) where the protein was truncated at N382. The minor βArr1 M411 resonance is only visible in the presence of enNTS$_1$ΔM4 (grey),

suggesting that it reflects a receptor-bound conformation. C) ^{1}H-^{13}C HMQC spectra of [$^{13}C^{\varepsilon}H_3$-methionine]-enNTS$_1$ΔM4 binary and βArr1-3A ternary complexes in the absence of PIP2. Overlay of NT8-13:enNTS$_1$ΔM4 (magenta) and NT8-13:enNTS$_1$ΔM4:βArr1-3A (cyan) ^{1}H-^{13}C HMQC spectra; asterisks indicate natural abundance βArr1-3A M411 peaks. Both spectra were recorded at 600 MHz with enNTS$_1$ΔM4 concentrations of 66 μM and 2.3x molar excess βArr1-3A was used in the NT8-13:enNTS$_1$ΔM4:βArr1-3A experiment.

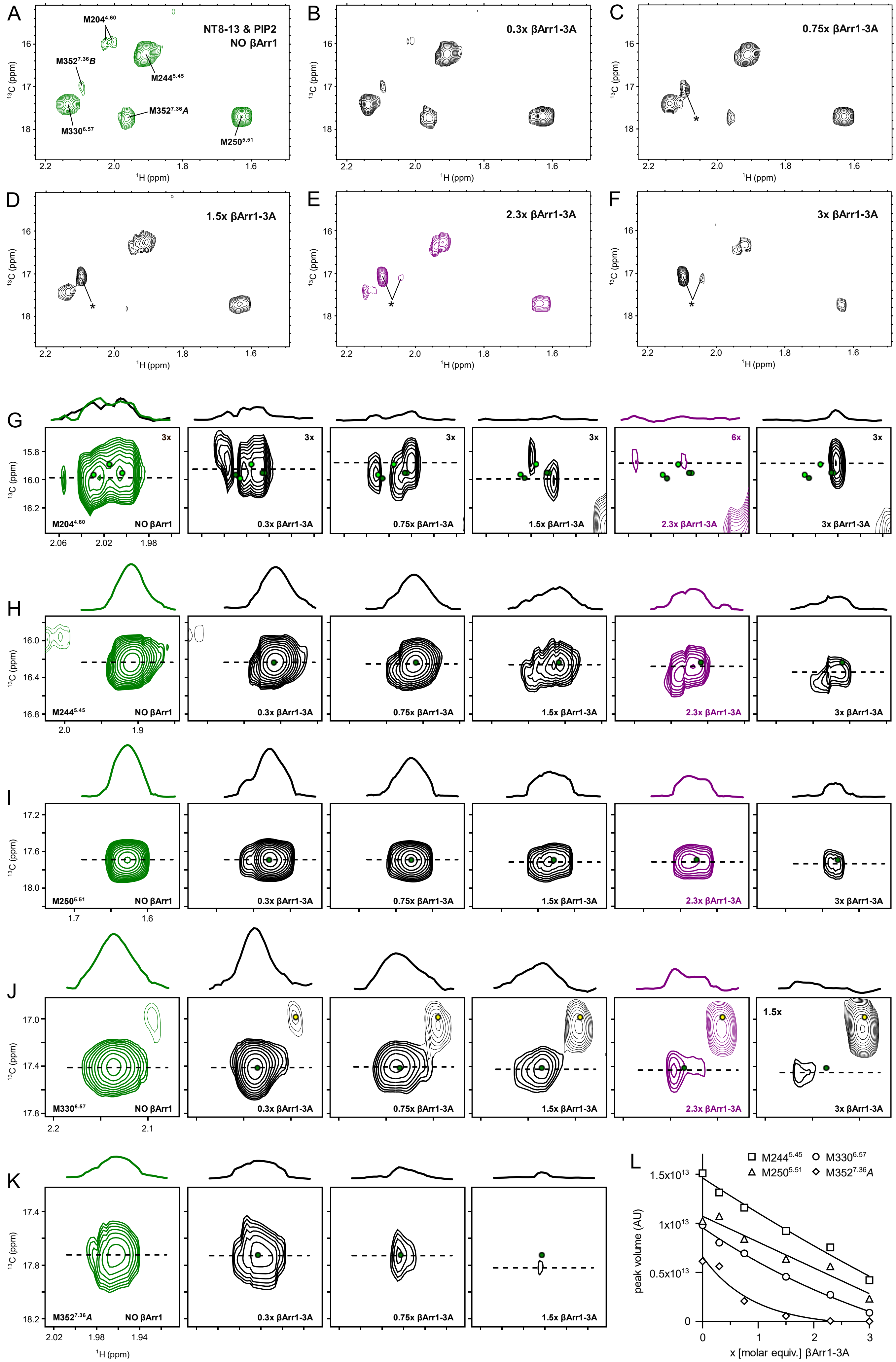
A
NT8-13 & PIP2
NO βArr1
M204 4.60
M352 7.36 B
M244 5.45
M352 7.36 A
M330 6.57
M250 5.51
B
0.3x βArr1-3A
C
0.75x βArr1-3A
D
1.5x βArr1-3A
E
2.3x βArr1-3A
F
3x βArr1-3A
13C (ppm)
1H (ppm)
G
H
I
J
K
NO βArr1
0.3x βArr1-3A
0.75x βArr1-3A
1.5x βArr1-3A
2.3x βArr1-3A
3x βArr1-3A
3x
6x
1.5x
L
M244 5.45
M250 5.51
M330 6.57
M352 7.36 A
peak volume (AU)
x [molar equiv.] βArr1-3A

Supplementary Figure 3. Titration of βArr1-3A into NT8-13:enNTS$_1$ΔM4:PIP2. ^{1}H-^{13}C HMQC spectra of enNTS$_1$ΔM4:NT8-13 in the presence of 0.0 (A), 0.3 (B), 0.75 (C), 1.5 (D), 2.3 (E), and 3.0 (F) molecular equivalents of βArr1-3A. Each spectrum was collected from separate, otherwise identical, NT8-13:enNTS$_1$ΔM4 samples. G-K) Extracted ^{1}H-^{13}C HMQC spectral regions for individual methionine resonances. 1D ^{1}H cross-sectional slices correspond to the dotted lines in the 2D spectra. All panels are plotted with identical contour levels unless otherwise indicated in the upper right corner (M204$^{4.60}$, 3x or 6x; M330$^{6.57}$, 1.5x). The resonances of other residues within the extracted region are drawn at 50% transparency. Dark green dots mark the positions of peaks in the absence βArr1-3A. Light green dots (G) indicate peak positions observed in an independent experiment of enNTS$_1$ΔM4:NT8-13 without transducer (Supplementary Figure 4A and green panels of Supplementary Figures 4G-K). Yellow dots (J) mark the position of M352$^{7.36}$B which is obscured by the major βArr1-3A M411 peak. L) Absolute peak volumes of selected residues plotted against molecular equivalents of βArr1-3A suggest peak broadening as a function of complex formation. Source data are provided as a Source Data file. All spectra were recorded at 600 MHz, in 3 mm thin wall precision NMR tubes (Wilmad), with enNTS$_1$ΔM4 concentrations of 66 μM.

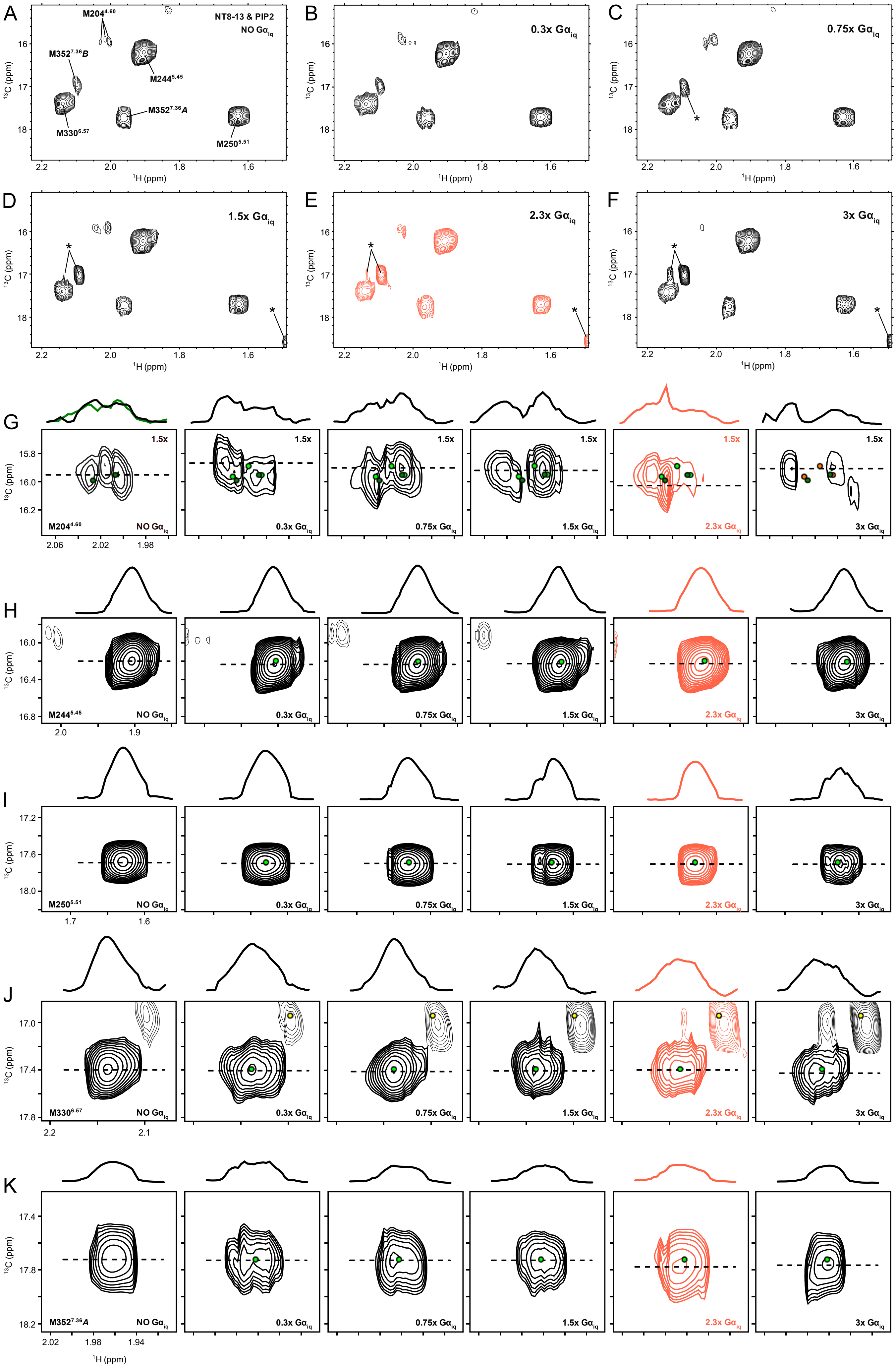

A
B
C
D
E
F
G
H
I
J
K
NT8-13 & PIP2
NO Gα$_{iq}$
0.3x Gα$_{iq}$
0.75x Gα$_{iq}$
1.5x Gα$_{iq}$
2.3x Gα$_{iq}$
3x Gα$_{iq}$
M204$^{4.60}$
M352$^{7.36}$B
M244$^{5.45}$
M352$^{7.36}$A
M330$^{6.57}$
M250$^{5.51}$
^{13}C (ppm)
^{1}H (ppm)
1.5x

Supplementary Figure 4. Titration of $G\alpha_{iq}$ into NT8-13:enNTS$_1$ΔM4. ^{1}H-^{13}C HMQC spectra of enNTS$_1$ΔM4:NT8-13 in the presence of 0.0 (A), 0.3 (B), 0.75 (C), 1.5 (D), 2.3 (E), and 3.0 (F) molecular equivalents of $G\alpha_{iq}$. Each spectrum was collected from separate, otherwise identical, NT8-13:enNTS$_1$ΔM4 samples. G-K) Extracted ^{1}H-^{13}C HMQC spectral regions for individual methionine resonances. 1D ^{1}H cross-sectional slices correspond to the dotted lines in the 2D spectra. All panels are plotted with identical contour levels unless otherwise indicated in the upper right corner (M204$^{4.60}$, 1.5x). The resonances of other residues within the extracted region are drawn at 50% transparency. Light green dots mark the positions of peaks in the absence of $G\alpha_{iq}$. Dark green dots (G) indicate peak positions observed in an independent experiment of NT8-13:enNTS$_1$ΔM4 without transducer (Supplementary Figure 3A and first panels of Supplementary Figures 3G-K). Yellow dots (J) mark the position of M352$^{7.36}$B which is obscured by the major natural abundance peak arising from $G\alpha_{iq}$. All spectra were recorded at 600 MHz, in 3 mm thin wall precision NMR tubes (Wilmad), with enNTS$_1$ΔM4 concentrations of 64 μM.

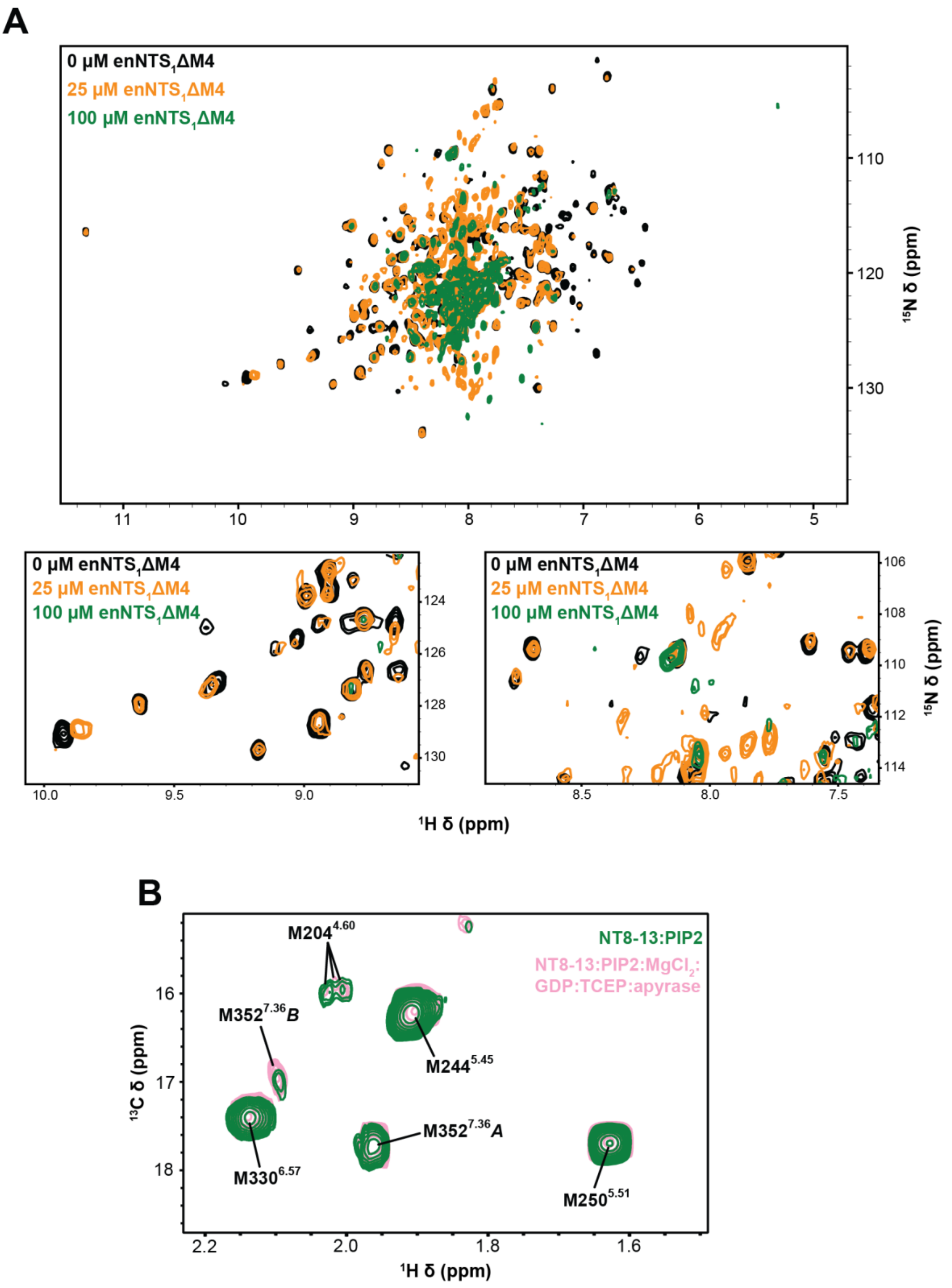

Supplementary Figure 5. Gα_{iq} forms a specific complex with NT8-13:enNTS$_1$ΔM4 that is unaffected by pyruvate and TCEP additives. A) ^{1}H-^{15}N TROSY-HSQC spectra overlay of 50 µM [*U*-^{15}N,^{13}C,^{2}H]-Gα_{iq} in the presence of 0 (black), 25 (orange) and 100 µM (green) unlabelled-enNTS$_1$ΔM4. Chemical shift perturbations and increasing peak intensities are observed for a subset of resonances consistent with a specific interaction despite the substantial increase in rotational correlation time and sub-optimal TROSY effect at 600 MHz field-strength. B) ^{1}H-^{13}C HMQC spectra of [^{13}C$^{\varepsilon}$H$_3$-methionine]-enNTS$_1$ΔM4:NT8-13:PIP2 in the presence (pink) and absence (green) of 2 mM MgCl2, 100 µM TCEP, 10 µM GDP and 0.25 units of apyrase. Both spectra were recorded at 600 MHz, in 3 mm thin wall precision NMR tubes (Wilmad), with enNTS$_1$ΔM4 concentrations of 66 µM.

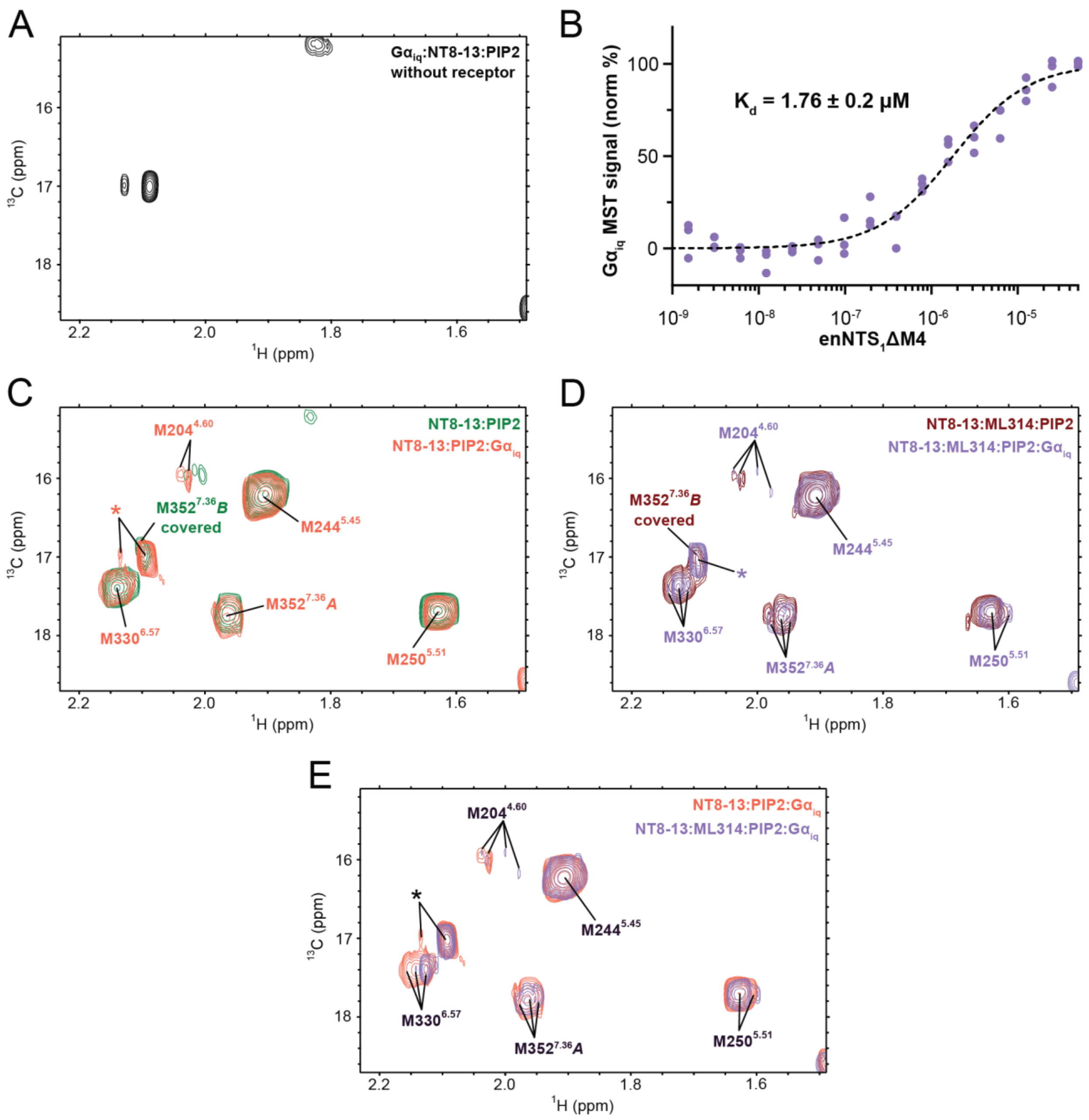

Supplementary Figure 6. The effect of various receptor and ligand combinations on Gα$_{iq}$ ^{1}H-^{13}C HMQC spectra and complex affinity. A) ^{1}H-^{13}C HMQC spectrum of 99 μM Gα$_{iq}$, without receptor, suggest natural abundance $^{13}CH_3$-methionine peaks arising from Gα$_{iq}$. B) Microscale thermophoresis (MST) measured the affinity of Gα$_{iq}$ for NT8-13:ML314:enNTS$_1$ΔM4 as 1.76 ± 0.2 μM (± SEM) using a single-site quadratic binding model. Data was collected as n=3 biologically independent experiments with 5 technical repeats. Source data are provided as a Source Data file. Overlays of NT8-13 (C) and NT8-13:ML314 (D) bound enNTS$_1$ΔM4 ^{1}H-^{13}C HMQC spectra with and without Gα$_{iq}$. E) Comparison of NT8-13:enNTS$_1$ΔM4:PIP2:Gα$_{iq}$ (salmon) and NT8-13:ML314:enNTS$_1$ΔM4:PIP2:Gα$_{iq}$ (light purple) ^{1}H-^{13}C HMQC spectra. Peaks marked with an asterisk represent natural abundance $^{13}CH_3$-methionine peaks arising from Gα$_{iq}$. All spectra were recorded at 600 MHz, in 3 mm thin wall precision NMR tubes (Wilmad), with enNTS$_1$ΔM4 concentrations of 64 μM.

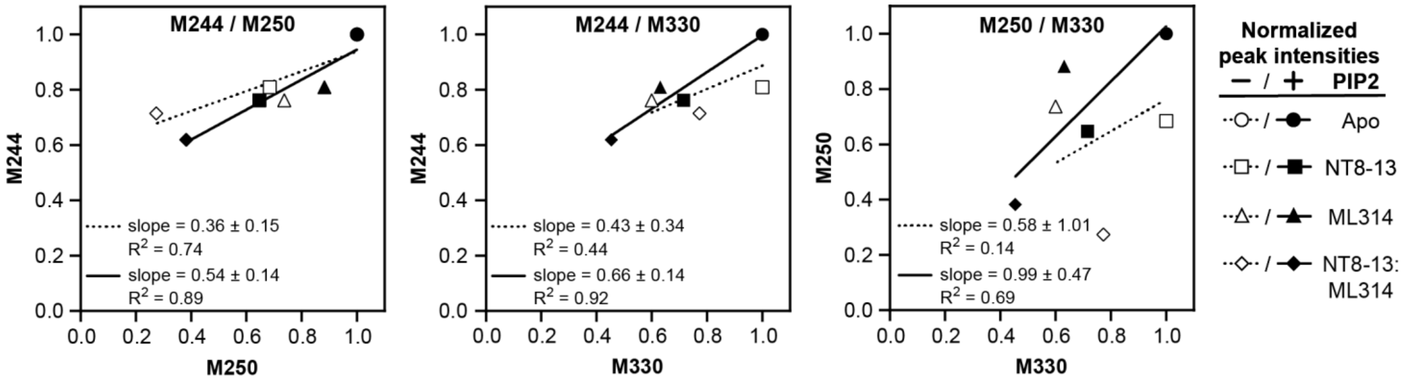

Supplementary Figure 7. PIP2 mediates long-range cooperativity between the orthosteric pocket and connector region. Pairwise correlation plots of normalized peak intensities (integrated peak volumes) for M244$^{5.45}$, M250$^{5.51}$, and M330$^{6.37}$ resonances. Symbols correspond to Apo (circle), NT8-13- (square), ML314- (triangle) and NT8-13 & ML314-bound (diamond) enNTS$_1$ΔM4 in the presence (filled symbol; solid line) and absence (empty symbol; dotted line) of PIP2. The corresponding slopes and R^2 values are indicated within each panel. Source data are provided as a Source Data file.

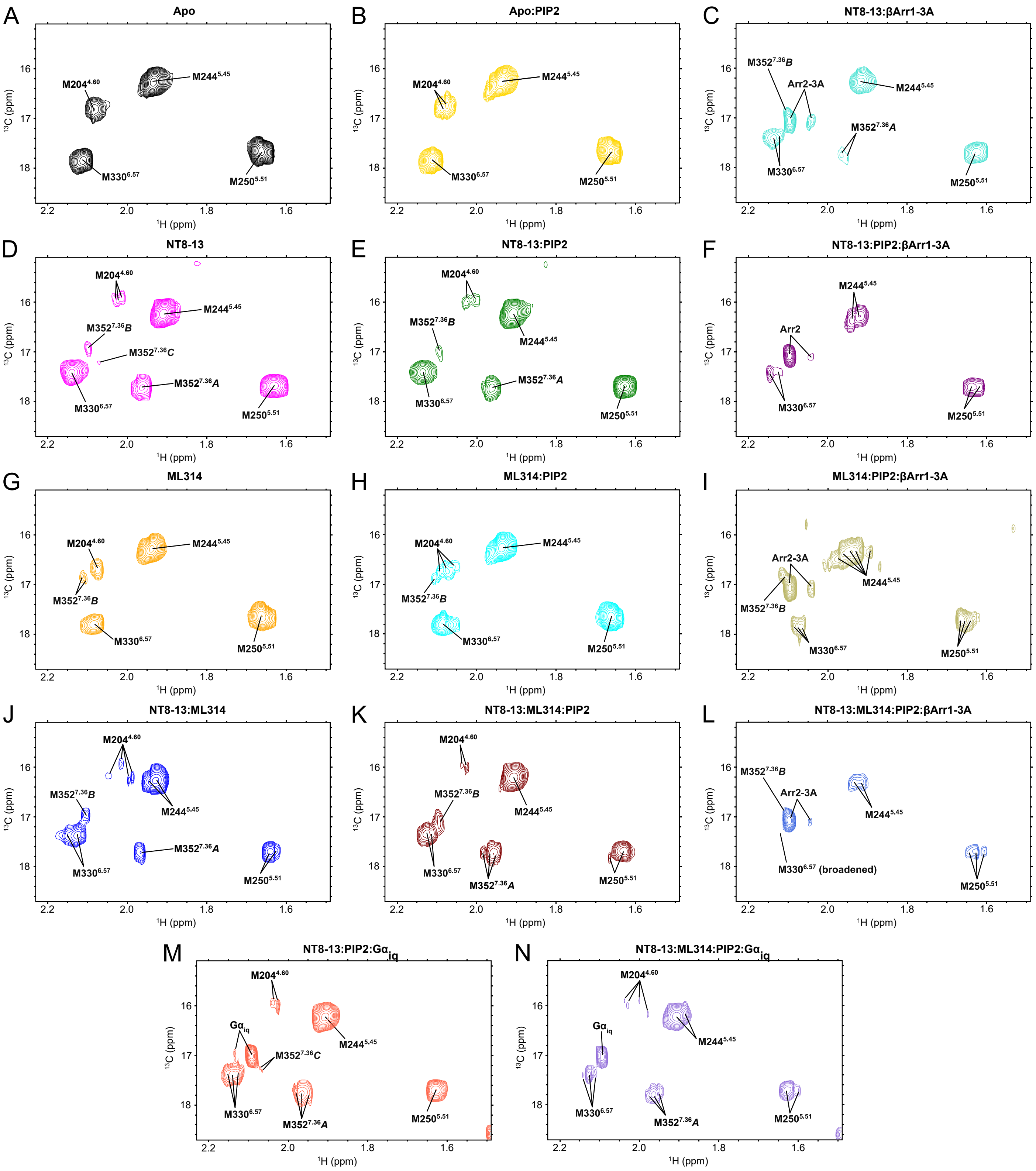

Supplementary Figure 8. ^{1}H-^{13}C HMQC spectra of enNTS$_1$ΔM4 ^{13}C$^{\epsilon}$H$_3$-methionine for all ligand:transducer protein conditions. Spectra shown are Apo (A), Apo:PIP2 (B), NT8-13:βArr1-3A (C), NT8-13 (D), NT8-13:PIP2 (E), NT8-13:PIP2:βArr1-3A (F), ML314 (G), ML314:PIP2 (H), ML314:PIP2:βArr1-3A (I), NT8-13:ML314 (J), NT8-13:ML314:PIP2 (K), NT8-13:ML314:PIP2:βArr1-3A (L), NT8-13:PIP2:Gαiq (M), and NT8-13:ML314:PIP2:Gα$_{iq}$ (N). Colour coding of individual spectra match those used throughout the manuscript. (F, I-O) Only spectra at 2.3x molar equivalents transducer are reproduced. All spectra were recorded at 600 MHz, in 3 mm thin wall precision NMR tubes (Wilmad), at enNTS$_1$ΔM4 concentrations of 66 μM (A-L) and 64 μM (M and N). Spectra A, D, G, and J were collected previously[1].

Supplementary References

1. Bumbak, F. et al. Ligands selectively tune the local and global motions of neurotensin receptor 1 (NTS1). *Cell Rep.* **42**, 112015 (2023).
