# Supplementary material for: Stabilization of pre-existing neurotensin receptor conformational states by β-arrestin-1 and the biased allosteric modulator ML314
Source: Nat Commun. 2023 Jun 7;14:3328. doi: 10.1038/s41467-023-38894-8 (PMC10247727; doi:10.1038/s41467-023-38894-8)
Supplement: Supplementary file 2 — Reporting Summary [file 41467_2023_38894_MOESM2_ESM.pdf]

nature portfolio

Corresponding author(s): Fabian Bumbak
Joshua Ziarek

Last updated by author(s): May 10, 2023

Reporting Summary

Nature Portfolio wishes to improve the reproducibility of the work that we publish. This form provides structure for consistency and transparency in reporting. For further information on Nature Portfolio policies, see our Editorial Policies and the Editorial Policy Checklist.

Statistics

For all statistical analyses, confirm that the following items are present in the figure legend, table legend, main text, or Methods section.

n/a	Confirmed	
☐	☒	The exact sample size (*n*) for each experimental group/condition, given as a discrete number and unit of measurement
☐	☒	A statement on whether measurements were taken from distinct samples or whether the same sample was measured repeatedly
☒	☐	The statistical test(s) used AND whether they are one- or two-sided<br>*Only common tests should be described solely by name; describe more complex techniques in the Methods section.*
☒	☐	A description of all covariates tested
☒	☐	A description of any assumptions or corrections, such as tests of normality and adjustment for multiple comparisons
☐	☒	A full description of the statistical parameters including central tendency (e.g. means) or other basic estimates (e.g. regression coefficient) AND variation (e.g. standard deviation) or associated estimates of uncertainty (e.g. confidence intervals)
☒	☐	For null hypothesis testing, the test statistic (e.g. *F*, *t*, *r*) with confidence intervals, effect sizes, degrees of freedom and *P* value noted<br>*Give P values as exact values whenever suitable.*
☒	☐	For Bayesian analysis, information on the choice of priors and Markov chain Monte Carlo settings
☒	☐	For hierarchical and complex designs, identification of the appropriate level for tests and full reporting of outcomes
☐	☒	Estimates of effect sizes (e.g. Cohen's *d*, Pearson's *r*), indicating how they were calculated

Our web collection on statistics for biologists contains articles on many of the points above.

Software and code

Policy information about availability of computer code

Data collection	NanoTemper Monolith NT (MST), Bruker Topspin v3.6.2 (NMR)
Data analysis	Sigma Plot v15 (MST), qMDD (1H-13C SOFAST-HMQC NMR), NMRPipe (NMR), NMRFAM Sparky v1.47 (NMR), GraphPad Prism v9.5.1 (NMR Peak Integrals)

For manuscripts utilizing custom algorithms or software that are central to the research but not yet described in published literature, software must be made available to editors and reviewers. We strongly encourage code deposition in a community repository (e.g. GitHub). See the Nature Portfolio guidelines for submitting code & software for further information.

Data

Policy information about availability of data

All manuscripts must include a data availability statement. This statement should provide the following information, where applicable:

- Accession codes, unique identifiers, or web links for publicly available datasets
- A description of any restrictions on data availability
- For clinical datasets or third party data, please ensure that the statement adheres to our policy

Source data used for graphs are provided with this paper. The chemical shift assignments of 13C-SOFAST-HMQC spectra generated in this study have been deposited in the Biological Magnetic Resonance Bank (BMRB) under accession codes 51908 [https://doi.org/10.13018/BMR51908] (PIP2:Apo-state-enNTS1ΔM4), 51909 [https://doi.org/10.13018/BMR51909] (PIP2:NT8-13:enNTS1ΔM), 51910 [https://doi.org/10.13018/BMR51910] (PIP2:ML314:enNTS1ΔM4), 51911 [https://doi.org/10.13018/BMR51911] (PIP2:NT8-13:ML314:enNTS1ΔM4), 51914 [https://doi.org/10.13018/BMR51914] (NT8-13:enNTS1ΔM4:βArr1-3A), 51915 [https://

doi.org/10.13018/BMR51915] (PIP2:NT8-13:enNTS1ΔM4:βArr1-3A), 51916 [https://doi.org/10.13018/BMR51916] (PIP2:ML314:enNTS1ΔM4:βArr1-3A), 51917 [https://doi.org/10.13018/BMR51917] (PIP2:NT8-13:ML314:enNTS1ΔM4:βArr1-3A), 51921 [https://doi.org/10.13018/BMR51921] (PIP2:NT8-13:enNTS1ΔM4:Gαiq), and 51927 [https://doi.org/10.13018/BMR51727] (PIP2:NT8-13:ML314:enNTS1ΔM4:Gαiq). 13C-SOFAST-HMQC spectra of Apo-state, NT8-13-, ML314-, and NT8-13 & ML314-bound enNTS1ΔM4 were generated in a previous study (https://doi.org/10.1016/j.celrep.2023.112015) and the chemical shift assignments were deposited in the BMRB under accession codes 51728 [https://doi.org/10.13018/BMR51728] (Apo-state-enNTS1ΔM4), 51735 [https://doi.org/10.13018/BMR51735] (NT8-13:enNTS1ΔM), 51737 [https://doi.org/10.13018/BMR51737] (ML314:enNTS1ΔM4), 51738 [https://doi.org/10.13018/BMR51738] (NT8-13:ML314:enNTS1ΔM4). The NMR spectra generated during and/or analyzed during the current study are available from the corresponding author on reasonable request. PDB files referenced in this manuscript are available from the RCSB Protein Data Bank: 4BWB [https://doi.org/10.2210/pdb4BWB/pdb] (HTGH4-ΔIC3:NT8-13), 6YVR [https://doi.org/10.2210/pdb6YVR/pdb] (NTSR1-H4X:SR142948A, and 6Z4Q [https://doi.org/10.2210/pdb6Z4Q/pdb] (NTSR1-H4X:NT8-13).

Human research participants

Policy information about studies involving human research participants and Sex and Gender in Research.

Reporting on sex and gender	N/A
Population characteristics	N/A
Recruitment	N/A
Ethics oversight	N/A

Note that full information on the approval of the study protocol must also be provided in the manuscript.

Field-specific reporting

Please select the one below that is the best fit for your research. If you are not sure, read the appropriate sections before making your selection.

☒ Life sciences ☐ Behavioural & social sciences ☐ Ecological, evolutionary & environmental sciences

For a reference copy of the document with all sections, see nature.com/documents/nr-reporting-summary-flat.pdf

Life sciences study design

All studies must disclose on these points even when the disclosure is negative.

Sample size	All NMR experiments, except the enNTS1:NT8-13:PIP2 which was duplicated, were collected without replication. MST experiments were collected in experimental triplicate (i.e. three separately prepared samples) and all data were fitted to quadratic binding model.
Data exclusions	Outliers excluded from MST experiments are highlighted in the Source Data File.
Replication	NMR spectra collected in duplicate were inspected for chemical shift differences. MST experiments were collected in (at least) experimental triplicate (i.e. three separately prepared samples) and all data were fitted to quadratic binding model. All replications were successful.
Randomization	Experiments were planned systematically and appropriate controls were employed. Randomization was not involved.
Blinding	Data in this study were generated and analyzed in an objective manner. Blinding was not necessary due to absence of bias.

Reporting for specific materials, systems and methods

We require information from authors about some types of materials, experimental systems and methods used in many studies. Here, indicate whether each material, system or method listed is relevant to your study. If you are not sure if a list item applies to your research, read the appropriate section before selecting a response.

Materials & experimental systems

n/a	Involved in the study	
☒	☐	Antibodies
☒	☐	Eukaryotic cell lines
☒	☐	Palaeontology and archaeology
☒	☐	Animals and other organisms
☒	☐	Clinical data
☒	☐	Dual use research of concern

Methods

n/a	Involved in the study	
☒	☐	ChIP-seq
☒	☐	Flow cytometry
☒	☐	MRI-based neuroimaging
